# Patient Judgments About Hypertension Control: The Role of Variability, Trends, and Outliers in Visualized Blood Pressure Data

**DOI:** 10.2196/11366

**Published:** 2019-03-26

**Authors:** Victoria Anne Shaffer, Pete Wegier, KD Valentine, Jeffery L Belden, Shannon M Canfield, Sonal J Patil, Mihail Popescu, Linsey M Steege, Akshay Jain, Richelle J Koopman

**Affiliations:** 1 University of Missouri Department of Psychological Sciences Columbia, MO United States; 2 Sinai Health System Temmy Latner Center for Palliative Care Toronto, ON Canada; 3 Sinai Health System Lunenfeld-Tanenbaum Research Institute Toronto, ON Canada; 4 University of Missouri Department of Family & Community Medicine Columbia, MO United States; 5 University of Missouri Department of Health Management & Informatics Columbia, MO United States; 6 University of Wisconsin School of Nursing Madison, WI United States; 7 University of Missouri Department of Electrical and Computer Engineering Columbia, MO United States

**Keywords:** data visualization, hypertension, hypertension control, patients’ judgment, primary care

## Abstract

**Background:**

Uncontrolled hypertension is a significant health problem in the United States, even though multiple drugs exist to effectively treat this chronic disease.

**Objective:**

As part of a larger project developing data visualizations to support shared decision making about hypertension treatment, we conducted a series of studies to understand how perceptions of hypertension control were impacted by data variations inherent in the visualization of blood pressure (BP) data.

**Methods:**

In 3 Web studies, participants (internet sample of patients with hypertension) reviewed a series of vignettes depicting patients with hypertension; each vignette included a graph of a patient’s BP. We examined how data visualizations that varied by BP mean and SD (Study 1), the pattern of change over time (Study 2), and the presence of extreme values (Study 3) affected patients’ judgments about hypertension control and the need for a medication change.

**Results:**

Participants’ judgments about hypertension control were significantly influenced by BP mean and SD (Study 1), data trends (whether BP was increasing or decreasing over time—Study 2), and extreme values (ie, outliers—Study 3).

**Conclusions:**

Patients’ judgment about hypertension control is influenced both by factors that are important predictors of hypertension related-health outcomes (eg, BP mean) and factors that are not (eg, variability and outliers). This study highlights the importance of developing data visualizations that direct attention toward clinically meaningful information.

## Introduction

Uncontrolled hypertension is a significant health problem; there are 75 million adults in the United States alone with diagnosed hypertension [1-3]. Hypertension control is an important goal in primary care because uncontrolled hypertension is a major risk factor for morbidity and mortality and contributes to heart disease, stroke, and chronic kidney disease [2]. Multiple drugs exist that effectively treat hypertension, yet hypertension remains uncontrolled in 46% of patients. Several national and regional health initiatives (eg, Healthy People 2020, Million Hearts Initiative, and Community Preventive Task Force) have focused on improving hypertension monitoring and management. However, despite these efforts, data from the National Health and Nutrition Examination Survey in 2011-2014 showed no marked change in the percentage of adults with controlled hypertension [4].

In their hypertension clinical action model, Kerr et al [5] identified 4 factors that predict uncontrolled hypertension—clinical uncertainty, competing demands/prioritization, medication-related factors (eg, side effects), and organizational factors (eg, lack of support to follow and reassess patients). However, the primary reason for failing to intensify medication in a clinic visit was uncertainty about the “true” blood pressure (BP) value. When multiple BP readings were recorded, there was often a discrepancy between the values with some readings falling inside the goal range and others falling outside, leading physicians and patients to question whether the action was warranted. BP measurements that occur both within and above the goal range can be especially perplexing to patients, leading to difficulty in making decisions about BP control [6-8].

To improve hypertension control in the primary care setting, our research team has developed a data visualization tool designed to support shared decision making about hypertension treatment. Our tool is a visual display of the patients’ BP data over the last 2 years that will be embedded in the electronic health record (EHR) and will be jointly viewed by patients and clinicians during a primary care office visit. This tool aims to (1) reduce clinical uncertainty about BP data and hypertension control; and (2) increase patients’ willingness to intensify their medication or comply with standing treatment plans, with the downstream benefit of improving hypertension control.

The data visualization tool was developed through a rapid prototyping process in which candidate visualizations were iteratively refined on the basis of regular feedback from patient and physician focus groups [9]. During the prototyping process, we concurrently conducted a series of vignette-based Web studies to inform the development of the data visualization tool. Previous research on the presentation of Web-based risk communications, including interactive graphics describing the risk of side effects for thyroid cancer treatments and EHR patient portal displays of laboratory blood test values, demonstrates the value of testing the effect of data visualization interventions on gist knowledge, perceptions of risk, and judgments about health status [10-13]. To date, no research has examined how data visualizations of BP designed for use in the EHR impact patients’ perceptions of risk and hypertension control. This paper aims to report the results of 3 studies that examined how data visualizations that varied in BP mean and SD (Study 1), pattern of change over time (Study 2), and the presence of extreme values (Study 3) impact patients’ judgments about hypertension control and the need for medication change.

## Methods

### Study Designs

Three demographically diverse internet samples of patients with hypertension reviewed several brief vignettes describing fictitious patients with hypertension through Web survey; each vignette included a graph of the patients’ BP data. All 3 studies used a within-subjects design, where all participants reviewed all vignettes, presented in random order, and provided judgments about the degree of hypertension control for every patient/vignette. The Web surveys are described below, and the results are reported in accordance with the Checklist for Reporting Results of Internet E-Surveys (ie, CHERRIES checklist) [14]. The Institutional Review Board at the University of Missouri approved all studies. The 3 studies had very similar methodologies; therefore, we will describe their methods and results together. All samples were recruited by Qualtrics, a survey company that maintains an opt-in demographically diverse internet panel that participates; details about participant recruitment are provided below.

**Figure 1:**
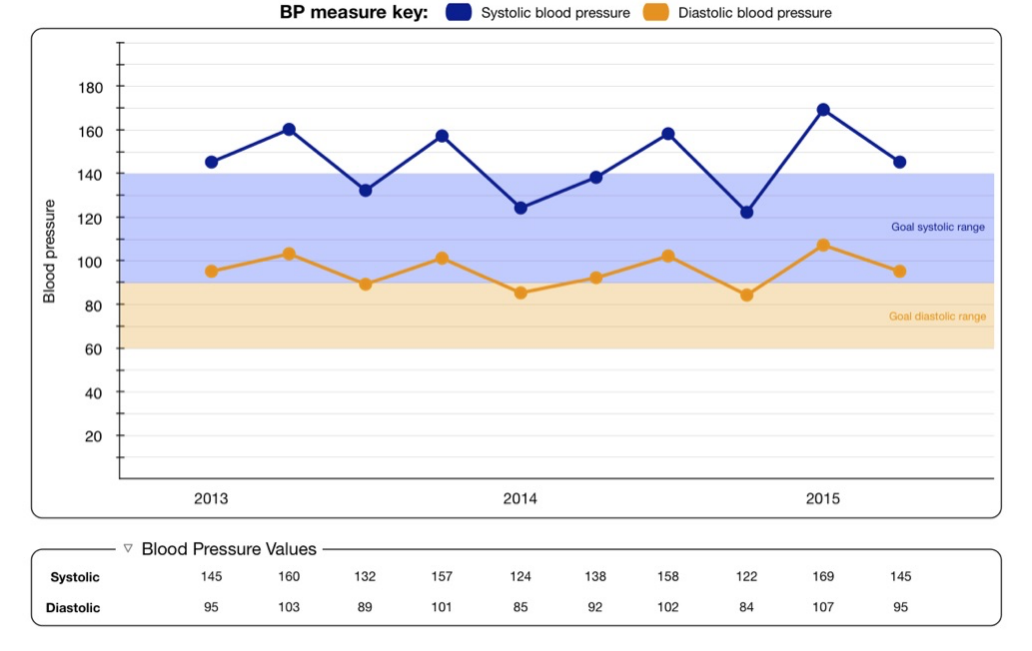
Sample visualization tool.

Each vignette described a patient who was being treated for hypertension and included a graph of the patient’s BP data over the past 2 years. Figure 1 presents an example of the data visualization tool. In Study 1, 9 vignettes systematically varied in the mean systolic BP (SBP; 130, 145, and 160 mm HG) and BP SD (5, 15, and 25) depicted in the graph; the slope was held constant (Multimedia Appendix 1). The mean SBP was chosen to represent clinical cases that included examples of controlled, uncontrolled, and borderline hypertension according to the 2014 Evidence-Based Guideline for the Management of High Blood Pressure in Adults from the panel members appointed to the Eighth Joint National Committee [15]. The SDs were chosen to represent small, moderate, and large mean variability according to published SBP values [16].

In Study 2, 6 vignettes differed in the SBP mean (130, 145, and 160 mm HG) and pattern of change over time (ie, trend or slope of the data; increasing or decreasing), while holding the SD constant (15 mm HG; Multimedia Appendix 2). In Study 3, we used 10 vignettes that differed in their mean SBP (130 and 145 mm HG), presence of outliers (no outliers, 1 outlier, or 2 outliers), and positioning of those outliers (above or below the mean; Multimedia Appendix 3).

### Outcomes

Primary outcomes were (1) perceived BP control; (2) need for medication change; (3) subjective risk of heart attack; and (4) subjective risk of stroke for each vignette. Perceived BP control and need for medication change were assessed using agree-disagree Likert scales, while the subjective likelihood of heart attack and stroke were measured using unlikely-likely Likert scales. In addition, we asked participants to estimate the proportion of SBP values out of range for each vignette: “What percentage of the patient’s systolic blood pressure values (ie, top number) would you estimate to be out of the goal range?”

After evaluating all vignettes, participants completed the Subjective Numeracy Scale (SNS) [17] and a Single-Item Literacy Screener for health literacy (SILS) [18]. In addition, participants provided demographic information (age, gender, race/ethnicity, education, and income) and responded to 2 additional items about how often they monitor and graph their own BP; no identifying information was collected or stored.

### Web Survey

All data were collected by open Web surveys developed using Qualtrics survey development software [19]. Informed consent, task instructions, practice task, vignettes, and study outcomes were all presented through a Web survey. The research team generated vignettes, BP displays, and outcomes for the studies. Of note, no adaptive items were used in the Web surveys. Responses to primary outcomes described above were recorded through slider bar. Responses to the SNS, SILS, and demographic items were all recorded using radio buttons on the Web survey, except age, which was recorded as free text. Responses to all items were required. Furthermore, back buttons on the browsers were disabled during the survey so participants could not go back and review previous webpages or change their responses on previous pages.

In all 3 Web surveys, participants completed one practice vignette that included directions for the practice task and 5 practice items; this section of the Web surveys utilized 3 webpages. Each vignette and its corresponding 5 questionnaire items (hypertension control, need for a medication change, 10-year heart attack risk, 10-year stroke risk, and percentage of SBP values out of goal range) added 2 additional pages to each Web survey (per vignette). The SILS and SNS scales added 9 items, and the demographic variables added 7 items to each Web study. Furthermore, we provided a free textbox at the end of the study for participants to share any additional feedback on the data visualizations presented in the Web studies.

In Study 1, the Web survey included 9 vignettes; therefore, participants completed 68 total items across 27 total webpages. In Study 2, the Web survey included 6 vignettes; participants completed 53 total items across 21 total webpages. In Study 3, the Web survey included 10 vignettes; participants completed 73 total items across 29 total webpages. The presentation order of the vignettes and their associated outcome measures was randomized within subjects. In addition, the team pilot-tested each Web survey themselves for functionality and usability before the surveys were deployed.

### Participant Recruitment

Qualtrics identified participants with hypertension through a single self-reported measure: “Has your doctor ever diagnosed you with hypertension, also known as high blood pressure?”; similar self-report items have been used to identify patients with hypertension in other epidemiological studies [20,21]. Informed consent was received from all participants after they were apprised about the purpose of the survey and the maximum completion time for the survey (30 minutes); participants were also provided with the name and contact information for the Project Director. Data for all 3 studies were collected between May 2016 and July 2016.

We used Internet Protocol addresses and Qualtrics identification numbers to determine unique site visitors. The survey completion rate was 86% (51/59) for Study 1, 79% (50/63) for Study 2, and 66% (55/83) for Study 3. All completed questionnaires were used in the analyses.

### Power and Statistical Analyses

We planned to recruit 50 patients with hypertension for each of the 3 Web studies. The sample size was determined *a priori* using G-Power [22,23] with the following data characteristics: 80% power to detect a significant effect (*f*=0.25) at an alpha of .05, with a minimum correlation of .50 between repeated measures. All outcomes were treated as continuous variables. We examined the effects of data variations on primary and secondary outcomes by conducting a series of multivariate analysis of variance tests for repeated measures. Predictors included the level of the mean SBP (all studies), SBP SD (Study 1), presence and direction of slope (Study 2), and the number and direction of outliers (Study 3). All tests were conducted in SPSS version 24 and R version 3.5 and were considered statistically significant when *P*<.05.

## Results

### Participants

Across the 3 experiments, a total of 156 patients with hypertension participated in this research. Participants were majority female (102/156, 65.4%) and white (116/156, 74.4%), with a mean age of 47.30 (range 19-79) years; Table 1 summarizes additional participants’ characteristics. In accordance with the sex and gender reporting guidelines [24], we present the data for all 3 studies disaggregated by gender in Multimedia Appendix 4.

In contrast, when the patient had BP that was objectively well controlled (mean 130 mm HG), the variability of the BP data had a significant impact on judgments about hypertension control and related health risks. For example, when the variability was low (SD 5 mm HG), BP was considered reasonably well controlled with little need for a medication change, and there was a much lower perceived risk of heart attack and stroke. In contrast, when variability in BP data was high (SD 25 mm HG), the perception of BP control was similar to those patients who had much higher mean (160 mm HG) BP values. This is noteworthy because evidence suggests that BP mean, much more than variability, is predictive of outcomes that matter to patients [25] (ie, heart attack and stroke). The effects of home BP variability on cardiovascular events and mortality are based on post-hoc analyses of 2 studies that looked at multiple indices of home BP variability [26]. The SD of BP is highly dependent on the mean BP, and it is unclear if indices of BP variability independent of the mean BP incrementally predict cardiovascular mortality or total mortality beyond mean SBP [27-29]. In addition, BP variability has unclear prognostic significance as varying methods or indices have been used to quantify the BP variability in all studies with no current standard or optimal indices available to quantify the BP variability.

**Figure 2:**
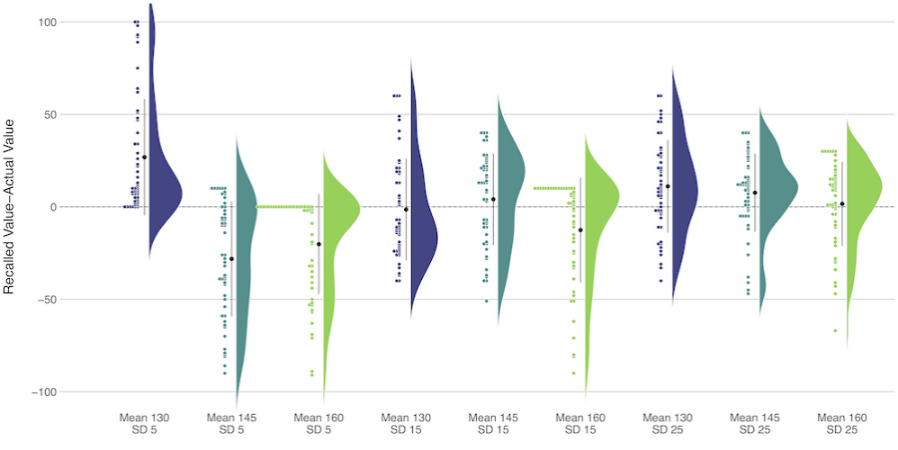
Error in the systolic blood pressure recall—Study 1, mean (large black dot) 95% CI.

Patients in Study 1 were also asked to recall the percentage of SBP values that were “out of range” (ie, exceeded 140 mm HG) in the graph. The recall was largely inaccurate but varied significantly by the SBP mean and SD (*F*_4,200_=8.73; *P*<.001; generalized η^2^=0.04; Figure 2). With low variability (SD 5), participants overestimated the percent of SBP values out of range when hypertension was controlled (mean 130) and underestimated the percent of SBP values out of range when hypertension was borderline (mean 145) or uncontrolled (mean 160). The estimates of SBP values out of range were more accurate when there was moderate to high variability in the BP data. Figure 2 shows a jellyfish plot of error in SBP recall. Dots above the dotted line represent participant overestimation, and dots below represent underestimation. For each vignette, we show the following: (1) the mean (large black dot) and 95% CI (black line) in the center; (2) a dot plot of all data points on the left; and (3) a kernel-density plot of the distribution of answers on the right.

### Study 1—Variations in SBP Mean and Variability

In Study 1, we observed a significant interaction between the SBP mean and SBP SD on perceived BP control (*F*_4,200_=16.94; *P*<.001; generalized η^2^=0.08), need for medication change (*F*_4,200_=16.19; *P*<.001; generalized η^2^=0.08), heart attack risk (*F*_4,200_=8.88; *P*<.001; generalized η^2^=0.04), and stroke risk (*F*_4,200_=11.70; *P*<.001; generalized η^2^=0.05; see Tables 2 and 3). When the mean SBP was high (160 mm HG), variability in the BP data did not impact the perception of BP control. Across all 3 SDs, participants reported that hypertension was *not* well controlled, patients *should* change their medication, and patients had an *elevated* risk of heart attack and stroke.

**Table:** 

Characteristics		Study 1, (n=51)	Study 2, (n=50)	Study 3, (n=53)
Gender, male, n (%)		19 (36)	17 (34)	18 (33)
**Age (years)**				
	Mean (SD)	47.0 (13.1)	44.7 (13.6)	50.2 (14.5)
	Range	19-72	20-79	19-77
**Race or ethnicity, n (%)**				
	White or Caucasian	44 (86)	34 (68)	38 (69)
	Black or African American	3 (6)	10 (20)	9 (16)
	Asian or Pacific Islander	2 (4)	1 (2)	3 (5)
	American Indian or Alaskan Native	0	1 (2)	1 (2)
	Hispanic or Latino/a	2 (4)	3 (6)	2 (4)
	Other	0	1 (2)	2 (4)
Single-Item Literacy Screener for health literacy, mean (SD)		2.0 (1.0)	1.8 (0.8)	2.0 (0.9)
Subjective Numeracy Scale, mean (SD)		4.3 (0.9)	4.3 (1.1)	4.3 (1.0)
**Education, n (%)**				
	Some high school	1 (2)	1 (2)	2 (4)
	High school graduate	10 (20)	14 (28)	13 (24)
	Some college	16 (32)	12 (24)	16 (29)
	Vocational training	4 (8)	7 (14)	4 (7)
	Associate’s degree	4 (8)	6 (12)	7 (13)
	Bachelor’s degree	9 (18)	5 (10)	8 (15)
	Master’s degree	0	3 (6)	4 (7)
	Professional degree	5 (10)	1 (2)	0
	Doctoral degree	1 (2)	1 (2)	1 (2)
**Income (US $), n (%)**				
	<10k	5 (10)	1 (2)	1 (2)
	10-19k	4 (8)	7 (14)	6 (11)
	20-29k	7 (13)	7 (14)	13 (24)
	30-39k	5 (10)	7 (14)	10 (18)
	40-49k	9 (17)	4 (8)	5 (9)
	50-59k	4 (8)	9 (18)	8 (15)
	60-69k	4 (8)	5 (10)	2 (4)
	70-79k	3 (6)	0	3 (5)
	80-89k	2 (4)	2 (4)	4 (7)
	90-99k	1 (2)	2 (4)	0
	100-149k	6 (12)	5 (10)	1 (2)
	>149k	1 (2)	1 (2)	2 (4)
**How often do you monitor your BP at home?, n (%)**				
	Never	11 (22)	7 (14)	8 (15)
	Annually	4 (8)	4 (8)	4 (7)
	Monthly	13 (25)	19 (38)	14 (25)
	Weekly	9 (18)	12 (24)	21 (38)
	Daily	14 (27)	8 (16)	8 (15)
**How often do you graph your home BP measurements?, n (%)**				
	Never	29 (57)	32 (64)	33 (60)
	Annually	4 (8)	3 (6)	3 (5)
	Monthly	6 (12)	6 (12)	9 (16)
	Weekly	6 (12)	7 (14)	7 (13)
	Daily	6 (12)	2 (4)	3 (5)

**Table 2:** Results of Study 1—blood pressure control.

Level of agreement with the following statements (0 “Strongly Disagree”-100 “Strongly Agree”)		SD 5, mean (95% CI)	SD 15, mean (95% CI)	SD 25, mean (95% CI)
**This patient’s blood pressure is well controlled**				
	Mean BP^a,b^=130	79.37 (73.38-85.36)	56.82 (48.87-64.78)	29.18 (21.47-36.88)
	Mean BP=145	39.57 (30.80-48.33)	23.45 (15.97-30.93)	21.8 (13.85-29.76)
	Mean BP=160	22.75 (14.51-30.98)	16.61 (9.44-23.77)	17.57 (10.28-24.85)
**This patient needs to change their medication**				
	Mean BP=130	29.75 (21.41-38.12)	47.39 (39.46-55.32)	71.27 (63.31-79.24)
	Mean BP=145	58.16 (49.39-66.92)	76.53 (69.19-83.87)	85.04 (79.42-90.66)
	Mean BP=160	86.47 (80.52-92.42)	85.25 (78.51-92.00)	83.43 (76.12-90.74)

^a^BP: blood pressure.

^b^All BPs provided in mm HG.

### Study 2—Data Trends

**Figure 3:**
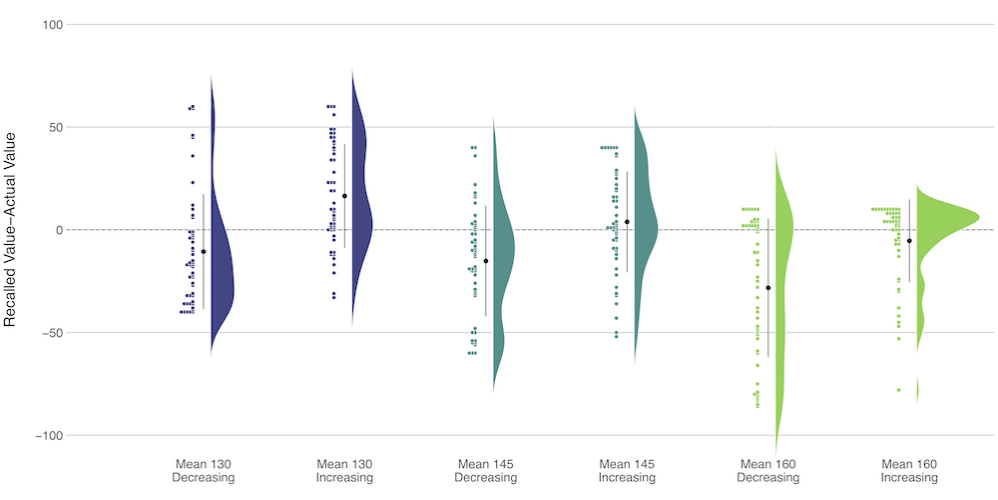
Error in the systolic blood pressure recall—Study 2, mean (large black dot) 95% CI.

In Study 2, we observed that patient ratings of hypertension control, need for medication changes, and 10-year risk of heart attack and stroke were significantly affected by trends visible in the BP data (see Tables 4 and 5) *.* Participants judged a positive data trend (depicting SBP values increasing over time) to be significantly less well controlled (*F*_1,49_=107.53; *P*<.001; generalized η^2^=0.45), in greater need of medication change (*F*_1,49_=129.07; *P*<.001; generalized η^2^=0.51), and at greater 10-year risk for a heart attack (*F*_1,49_=112.79; *P*<.001; generalized η^2^=0.47) and stroke (*F*_1,49_=111.52; *P*<.001; generalized η^2^=0.47) than a negative data trend (depicting SBP values decreasing over time) with the same SBP mean. Consistent with Study 1, participants’ recall of the percentage of SBP measurements out of range was largely inaccurate (Figure 3). Although the graphs depicting an increasing and decreasing trend had the same number of out-of-range measurements, participants recalled seeing a greater percentage of out-of-range values when the SBP was increasing over time than when the slope was decreasing (*F*_1,49_=33.04; *P*<.001; generalized η^2^=0.16).

### Study 3—Outliers

Another way of examining the effect of variability in judgments about BP control is to consider the presence of extreme values (ie, outliers) independent of the overall measurement variance. In Study 3, we compared judgments about uniform data patterns (ie, no discernable outliers) with those with either 1 or 2 outliers. In addition, we systematically varied whether those outliers were above or below the mean BP of the depicted time period. The presence and number of outliers significantly affected judgments of hypertension control (*F*_4, 216_=17.98; *P*<.001; generalized η^2^=0.14), need for medication change (*F*_4,216_=13.38; *P*<.001; generalized η^2^=0.11), and perceived risk of heart attack (*F*_4,216_=12.85; *P*<.001; generalized η^2^=0.10), and stroke (*F*_4,216_=13.81; *P*<.001; generalized η^2^=0.11; Tables 6 and 7). When any extreme values were present, participants judged the patient to have hypertension that was significantly less well controlled, to be in greater need of medication change, and to be at greater 10-year risk for a heart attack or stroke than when the data had a more uniform distribution. Furthermore, 2 outliers (whether above or below the mean) were considered significantly more concerning than a single outlier.

**Figure 4:**
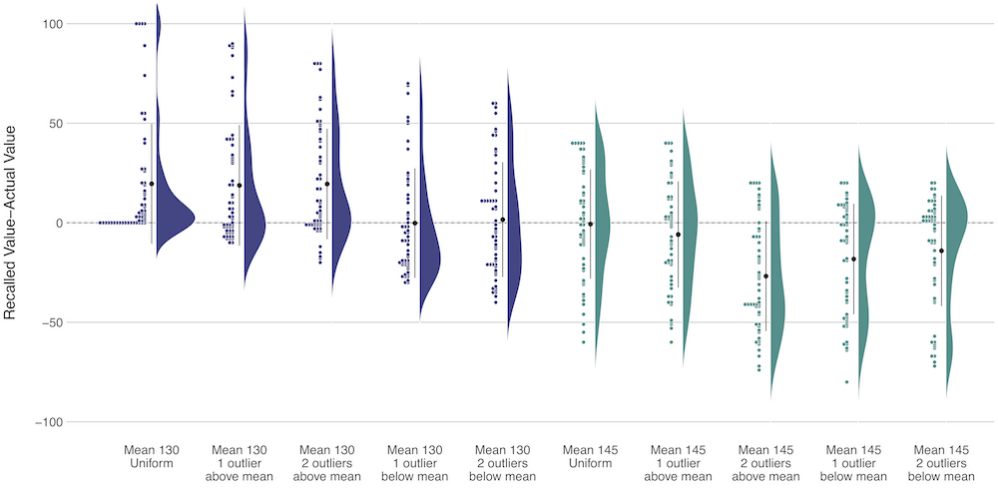
Error in the systolic blood pressure recall—Study 3, mean (large black dot) 95% CI.

Finally, recall for the percent of SBP values out of range was also significantly impacted by the presence and magnitude of extreme values (*F*_4,216_=5.54; *P*<.001; generalized η^2^=0.04; Figure 4). When hypertension was controlled (mean 130), participants overestimated the percent of SBP values out of range with a uniform distribution and outlier(s) above the mean but more accurately recalled the percent of SBP values out of range when the outlier(s) were below the mean. In contrast, with borderline hypertension control (mean 145), recall for percent of SBP values out of range was fairly accurate with a uniform distribution and when there was only one outlier above the mean. However, participants consistently underestimated the percent of SBP values out of range when there was a single outlier below the mean or 2 outliers in either direction.

**Table 3:** Results of Study 1—risk perception and recall.

Perceived likelihood of the following events (1 “Extremely Unlikely”-10 “Extremely Likely”)		SD 5, mean (95% CI)	SD 15, mean (95% CI)	SD 25, mean (95% CI)
**Heart attack in the next 10 years**				
	Mean BP^a,b^=130	3.82 (3.26-4.39)	4.29 (3.67-4.92)	6.33 (5.73-6.94)
	Mean BP=145	5.59 (4.90-6.27)	7.22 (6.57-7.86)	7.61 (7.09-8.13)
	Mean BP=160	7.67 (7.05-8.28)	7.98 (7.32-8.64)	8.12 (7.65-8.59)
**Stroke in the next 10 years**				
	Mean BP=130	3.76 (3.17-4.36)	4.45 (3.77-5.13)	6.63 (5.95-7.30)
	Mean BP=145	5.76 (5.08-6.45)	7.43 (6.84-8.03)	8.00 (7.45-8.62)
	Mean BP=160	8.04 (7.45-8.62)	8.14 (7.49-8.78)	8.35 (7.91-8.80)
**% of systolic blood pressure points out of range (0%-100%)**				
	Mean BP=130 (Actual)	0	40	40
	Mean BP=130 (Recalled)	26.84 (17.98-35.71)	38.53 (30.83-46.23)	51.06 (44.01-58.10)
	Mean BP=145 (Actual)	90	60	60
	Mean BP=145 (Recalled)	61.84 (53.10-70.59)	64.08 (57.12-71.04)	67.63 (61.69-73.57)
	Mean BP=160 (Actual)	100	90	70
	Mean BP=160 (Recalled)	79.78 (72.17-87.39)	77.42 (69.45-85.42)	71.63 (65.24-78.02)

^a^BP: blood pressure.

^b^All BPs provided in mm HG.

**Table 4:** Results of Study 2—blood pressure control.

Level of agreement with the following statements (0 “Strongly Disagree”-100 “Strongly Agree”)		Increasing, mean (95% CI)	Decreasing, mean (95% CI)
**This patient’s blood pressure is well controlled**			
	Mean BP^a,b^=130	30.88 (21.93-39.83)	85.72 (79.2-92.24)
	Mean BP=145	20.40 (13.06-27.74)	73.80 (66.18-81.42)
	Mean BP=160	11.26 (4.90-1762)	49.86 (40.45-59.27)
**This patient needs to change their medication**			
	Mean BP=130	82.26 (75.85-88.67)	21.70 (13.47-29.93)
	Mean BP=145	85.78 (79.37-92.19)	27.32 (19.41-35.23)
	Mean BP=160	90.80 (84.15-97.45)	45.50 (35.72-55.28)

^a^BP: blood pressure.

^b^All BPs provided in mm HG.

**Table 5:** Results of Study 2—risk perception and recall.

Perceived likelihood of the following events (1 “Extremely Unlikely”-10 “Extremely Likely)		Increasing, mean (95% CI)	Decreasing, mean (95% CI)
**Heart attack in the next 10 years**			
	Mean BP^a,b^=130	7.18 (6.58-7.78)	3.02 (2.42-3.62)
	Mean BP=145	7.96 (7.39-8.53)	3.66 (3.01-4.31)
	Mean BP=160	9.00 (8.56-9.44)	5.60 (4.89-6.31)
**Stroke in the next 10 years**			
	Mean BP=130	7.18 (6.55-7.81)	2.96 (2.34-3.58)
	Mean BP=145	7.98 (7.38-8.58)	3.60 (2.98-4.22)
	Mean BP=160	9.08 (8.60-9.56)	5.56 (4.80-6.32)
**% of systolic blood pressure points out of range (0%-100%)**			
	Mean BP=130 (Actual)>	40	40
	Mean BP=130 (Estimate)	56.48 (49.30-63.66)	29.4 (21.45-37.35)
	Mean BP=145 (Actual)	60	60
	Mean BP=145 (Estimate)	63.86 (56.89-70.83)	44.82 (37.15-52.49)
	Mean BP=160 (Actual)	90	90
	Mean BP=160 (Estimate)	84.66 (78.94-90.38)	61.78 (52.23-71.33)

^a^BP: blood pressure.

^b^All BPs provided in mm HG.

**Table 6:** Results of Study 3— blood pressure control.

Level of agreement with the following statements (0 “Strongly Disagree”-100 “Strongly Agree”)		Uniform, mean (95% CI)	1 Up, mean (95% CI)	1 Down, mean (95% CI)	2 Up, mean (95% CI)	2 Down, mean (95% CI)
**This patient’s blood pressure is well controlled**						
	Mean BP^a,b^ =130	89.22 (84.12-94.32)	68.91 (61.41-76.41)	67.71 (59.49-75.93)	53.07 (44.47-61.68)	47.02 (38.60-55.44)
	Mean BP=145	37.04 (28.15-45.92)	40.65 (32.40-48.91)	28.49 (20.33-36.65)	39.27 (30.43-48.12)	19.09 (11.84-26.34)
**This patient needs to change their medication**						
	Mean BP=130	20.87 (12.67-29.07)	33.18 (24.89-41.48)	32.47 (24.61-40.33)	49.87 (40.89-58.85)	53.98 (46.44-61.52)
	Mean BP=145	70.93 (62.51-79.35)	65.04 (56.26-73.81)	76.93 (69.52-84.34)	73.25 (65.42-81.09)	84.51 (77.91-91.11)

^a^BP: blood pressure.

^b^All BPs provided in mm HG.

**Table 7:** Results of Study 3—risk perception and recall.

Perceived likelihood of the following events (1 “Extremely Unlikely”-10 “Extremely Likely”)		Uniform, mean (95% CI)	1 Up, mean (95% CI)	1 Down, mean (95% CI)	2 Up, mean (95% CI)	2 Down, mean (95% CI)
**Heart attack in the next 10 years**						
	Mean BP^a,b^=130	2.65 (2.12-3.19)	4.02 (3.32-4.72)	3.87 (3.28-4.46)	5.29 (4.57-6.01)	4.85 (4.22-5.49)
	Mean BP=145	6.42 (5.75-7.08)	6.24 (5.63-6.84)	7.07 (6.48-7.66)	6.82 (6.10-7.54)	7.69 (7.11-8.27)
**Stroke in the next 10 years**						
	Mean BP=130	2.58 (2.08-3.08)	4.14 (3.40-4.88)	3.90 (3.24-4.56)	5.46 (4.69-6.23)	4.76 (4.06-5.46)
	Mean BP=145	6.55 (5.87-7.22)	6.42 (5.83-7.01)	7.35 (6.73-7.97)	6.95 (6.22-7.67)	7.98 (7.39-8.57)
**% of systolic blood pressure points out of range (0%-100%)**						
	Mean BP=130 (Actual)	0	10	30	20	40
	Mean BP=130 (Estimate)	19.58 (11.44-27.72)	28.80 (20.61-36.99)	29.27 (21.89-36.65)	39.47 (31.96-46.98)	41.58 (33.79-49.38)
	Mean BP=145 (Actual)	60	60	80	80	80
	Mean BP=145 (Estimate)	59.33 (51.92-66.74)	54.09 (46.88-61.31)	61.78 (54.28-69.29)	53.18 (45.69-60.67)	65.91 (58.41-73.41)

^a^BP: blood pressure.

^b^All BPs provided in mm HG.

## Discussion

### Principal Findings

To inform the development of a physician-patient shared data visualization tool for BP values, we conducted 3 vignette-based Web studies to understand better how patients interpret the variability in visualizations of BP data. In all studies, we observed that patients with hypertension consistently judged variations in BP data as meaningful indicators of hypertension control. In Study 1, increased variability in SBP data was associated with increasingly negative judgments about hypertension control, even when the mean SBP remained constant and within goal range. In Study 2, we demonstrated that the pattern of change in SBP values over time also significantly influenced judgments about hypertension control. While graphs depicting an increase in the SBP over time were appropriately judged to be of greater concern than graphs showing a decrease in the SBP over time, recall for the number of out-of-range BP values was inaccurate. When the SBP values increased over time, patients remembered more out-of-range values than when the slope decreased, even though the number of out-of-range values did not differ in our study. Finally, the presence of outliers (Study 3) also significantly impacted patients’ judgments about hypertension control, the need for a medication change, and the risks associated with uncontrolled hypertension. Observing even 1 or 2 outliers in BP data leads patients to inappropriately determine that hypertension is not well controlled.

The use of data visualization in electronic medical records has the potential to transform clinical encounters. While the technology to develop these tools is available, little is known about how these data displays will influence patients’ judgments about hypertension control and subsequent decisions regarding treatment. These 3 studies have demonstrated that judgments about hypertension control are strongly and inappropriately influenced by the presence of outliers and variability in the data. Outliers and variability mask mean BP values and the presence of data trends—important predictors of BP-related health outcomes (eg, heart attack or stroke) [25]. These findings are important for the development of interventions to promote shared decision making in primary care, which must direct attention to clinically meaningful information, that is, mean BP and trends rather than variability in BP or outliers [25]. It should be stressed that these conclusions about the potential benefits of visualization techniques that minimize the impact of outliers and variability would apply only to measurements obtained from well-calibrated BP measurement devices. These recommendations should not be applied to devices where variability is derived from inaccurate measurement.

### Limitations

There are several limitations to these studies that potentially constrain their generalizability. One limitation is the use of internet patient samples. When patients are making decisions about treatment for hypertension, they are typically made in conjunction with their physician during a clinic visit. In addition, while our sample of patients is more demographically diverse than typical internet samples, it is not representative of the population of patients with hypertension. Furthermore, we focused only on patients in these studies; therefore, future work should examine the effect of data visualization on physicians’ judgments about hypertension control, as well as the effect on shared patient-physician decisions. It is possible that physicians will perform similarly to patients because we are examining judgments that stem from common perceptual and cognitive processes [30,31]. On the other hand, physicians may have greater knowledge about the relative importance of the BP mean and variability than patients, which could alter their judgments.

### Conclusions

Health information technologies provide an opportunity for patients to become more engaged in decision making about hypertension control. We are endeavoring to design a data visualization tool for BP that can be jointly used by physicians and patients in this decision-making process. This tool aims to make the limited time shared in the exam room more efficient and effective. Defining how data elements, such as trends, variability, and outliers, support or detract from an understanding of the data will aid in the design of data visualizations that highlight meaningful characteristics of the data; this may, in turn, result in shared decisions that are better informed. Areas for future study include understanding how these parameters influence physician judgments about hypertension control and how information acquisition from data visualizations is affected by numeracy, health literacy, and graph literacy of patients and physicians.

## Appendix

Multimedia Appendix 1 Study 1 Materials.

Multimedia Appendix 2 Study 2 Materials.

Multimedia Appendix 3 Study 3 Materials.

Multimedia Appendix 4 Data for Studies 1-3 disaggregated by gender.

## Abbreviations

BP: blood pressure
EHR: electronic health record
SILS: Single-Item Literacy Screener for health literacy
SBP: systolic blood pressure
SNS: Subjective Numeracy Scale

## References

[1] Go A, Mozaffarian D, Roger V, Benjamin Emelia J, Berry Jarett D, Borden William B, Bravata Dawn M, Dai Shifan, Ford Earl S, Fox Caroline S, Franco Sheila, Fullerton Heather J, Gillespie Cathleen, Hailpern Susan M, Heit John A, Howard Virginia J, Huffman Mark D, Kissela Brett M, Kittner Steven J, Lackland Daniel T, Lichtman Judith H, Lisabeth Lynda D, Magid David, Marcus Gregory M, Marelli Ariane, Matchar David B, McGuire Darren K, Mohler Emile R, Moy Claudia S, Mussolino Michael E, Nichol Graham, Paynter Nina P, Schreiner Pamela J, Sorlie Paul D, Stein Joel, Turan Tanya N, Virani Salim S, Wong Nathan D, Woo Daniel, Turner Melanie B, American Heart Association Statistics Committee and Stroke Statistics Subcommittee (2013). Heart disease and stroke statistics--2013 update: a report from the American Heart Association. Circulation.

[2] Roger V, Go A, Lloyd-Jones D (2012). Heart disease and stroke statistics--.

[3] Merai R, Siegel C, Rakotz M, Basch P, Wright J, Wong B, Thorpe P, DHSc (2016). CDC Grand Rounds: A Public Health Approach to Detect and Control Hypertension. MMWR Morb Mortal Wkly Rep.

[4] Yoon SSS, Carroll MD, Fryar CD (2015). Hypertension Prevalence and Control Among Adults: United States, 2011-2014. NCHS Data Brief.

[5] Kerr Ea, Zikmund-Fisher B, Klamerus M, Subramanian U, Hogan M, Hofer Timothy P (2008). The role of clinical uncertainty in treatment decisions for diabetic patients with uncontrolled blood pressure. Ann Intern Med.

[6] Lebeau J, Cadwallader J, Aubin-Auger I, Mercier A, Pasquet T, Rusch E, Hendrickx K, Vermeire E (2014). The concept and definition of therapeutic inertia in hypertension in primary care: a qualitative systematic review. BMC Fam Pract.

[7] Crowley MJ, Smith VA, Olsen MK, Danus S, Oddone EZ, Bosworth HB, Powers BJ (2011). Treatment intensification in a hypertension telemanagement trial: clinical inertia or good clinical judgment?. Hypertension.

[8] Kolata G (2015). The New York Times.

[9] Wegier P, Belden J, Canfield S (2016). Designing data visualizations to support shared decision making about blood pressure.

[10] Zikmund-Fisher BJ, Exe NL, Witteman HO (2014). Numeracy and literacy independently predict patients' ability to identify out-of-range test results. J Med Internet Res.

[11] Zikmund-Fisher B, Witteman H, Fuhrel-Forbis A, Exe N, Kahn V, Dickson M (2012). Animated graphics for comparing two risks: a cautionary tale. J Med Internet Res.

[12] Zikmund-Fisher BJ, Dickson M, Witteman HO (2011). Cool but counterproductive: interactive, Web-based risk communications can backfire. J Med Internet Res.

[13] Zikmund-Fisher B, Scherer A, Witteman H, Solomon J, Exe N, Fagerlin A (2018). Effect of Harm Anchors in Visual Displays of Test Results on Patient Perceptions of Urgency About Near-Normal Values: Experimental Study. J Med Internet Res.

[14] Eysenbach G (2004). Improving the quality of Web surveys: the Checklist for Reporting Results of Internet E-Surveys (CHERRIES). J Med Internet Res.

[15] James PA, Oparil S, Carter BL, Cushman WC, Dennison-Himmelfarb C, Handler J, Lackland DT, LeFevre ML, MacKenzie TD, Ogedegbe O, Smith SC, Svetkey LP, Taler SJ, Townsend RR, Wright JT, Narva AS, Ortiz E (2014). 2014 evidence-based guideline for the management of high blood pressure in adults: report from the panel members appointed to the Eighth Joint National Committee (JNC 8). JAMA.

[16] Juhanoja EP, Niiranen TJ, Johansson JK, Puukka PJ, Jula AM (2016). Agreement between ambulatory, home, and office blood pressure variability. J Hypertens.

[17] Fagerlin A, Zikmund-Fisher BJ, Ubel PA, Jankovic A, Derry HA, Smith DM (2007). Measuring numeracy without a math test: development of the Subjective Numeracy Scale. Med Decis Making.

[18] Morris NS, MacLean CD, Chew LD, Littenberg B (2006). The Single Item Literacy Screener: evaluation of a brief instrument to identify limited reading ability. BMC Fam Pract.

[19] (2018). Qualtrics.

[20] Centers for Disease ControlPrevention (CDC) (2012). Vital signs: awareness and treatment of uncontrolled hypertension among adults--United States, 2003-2010. MMWR Morb Mortal Wkly Rep.

[21] Diaz VA, Mainous AG, Koopman RJ, Geesey ME (2004). Undiagnosed obesity: implications for undiagnosed hypertension, diabetes, and hypercholesterolemia. Fam Med.

[22] Faul F, Erdfelder E, Buchner A, Lang A (2009). Statistical power analyses using G*Power 3.1: tests for correlation and regression analyses. Behav Res Methods.

[23] Faul F, Erdfelder E, Lang A, Buchner Axel (2007). G*Power 3: a flexible statistical power analysis program for the social, behavioral, and biomedical sciences. Behav Res Methods.

[24] Heidari S, Babor TF, De Castro P, Tort S, Curno M (2018). [Sex and gender equity in research: rationale for the SAGER guidelines and recommended use]. Gac Sanit.

[25] Hansen TW, Thijs L, Li Y, Boggia J, Kikuya M, Björklund-Bodegård K, Richart T, Ohkubo T, Jeppesen J, Torp-Pedersen C, Dolan E, Kuznetsova T, Stolarz-Skrzypek K, Tikhonoff V, Malyutina S, Casiglia E, Nikitin Y, Lind L, Sandoya E, Kawecka-Jaszcz K, Imai Y, Wang J, Ibsen H, O'Brien E, Staessen JA, International Database on Ambulatory Blood Pressure in Relation to Cardiovascular Outcomes Investigators (2010). Prognostic value of reading-to-reading blood pressure variability over 24 hours in 8938 subjects from 11 populations. Hypertension.

[26] Stergiou GS, Ntineri A, Kollias A, Ohkubo T, Imai Y, Parati G (2014). Blood pressure variability assessed by home measurements: a systematic review. Hypertens Res.

[27] Asayama K, Kikuya M, Schutte R, Thijs L, Hosaka M, Satoh M, Hara A, Obara T, Inoue R, Metoki H, Hirose T, Ohkubo T, Staessen JA, Imai Y (2013). Home blood pressure variability as cardiovascular risk factor in the population of Ohasama. Hypertension.

[28] Rothwell PM, Howard SC, Dolan E, O'Brien E, Dobson JE, Dahlöf B, Poulter NR, Sever PS, ASCOT-BPLAMRC Trial Investigators (2010). Effects of beta blockers and calcium-channel blockers on within-individual variability in blood pressure and risk of stroke. Lancet Neurol.

[29] Diaz KM, Tanner RM, Falzon L, Levitan EB, Reynolds K, Shimbo D, Muntner P (2014). Visit-to-visit variability of blood pressure and cardiovascular disease and all-cause mortality: a systematic review and meta-analysis. Hypertension.

[30] (2014). Belden J, Lowrance N, Patel J.

[31] Ware C (2005). Visual Queries: The Foundation of Visual Thinking. Knowledge and Information Visualization.

