# Supplementary material for: Patient Judgments About Hypertension Control: The Role of Variability, Trends, and Outliers in Visualized Blood Pressure Data
Source: J Med Internet Res. 2019 Mar 26;21(3):e11366. doi: 10.2196/11366 (PMC6454346; doi:10.2196/11366)
Supplement: Multimedia Appendix 1 [file jmir_v21i3e11366_app1.pdf]

Multimedia Appendix A: Study 1 Materials

Text for each vignette:

Mr. Brown visits his primary care physician and has his blood pressure measured. Mr. Brown and his doctor examine his blood pressure control over the last two years. Below is a graph showing his systolic and diastolic blood pressure over two years. After reviewing the graph, we will ask you several questions about Mr. Brown and his blood pressure data. For this task, you will need to know what normal values are for blood pressure. When you are resting, a normal systolic value (i.e. the top number) should be between 100 and 140 mmHg and a normal diastolic value (i.e. the bottom number) will be between 60 and 90mmHg. [The only variation in the text of the vignettes was the patient name. 9 total vignettes were presented in Study 1]

Vignette 1 (SBP Mean 130, SBP SD 5)

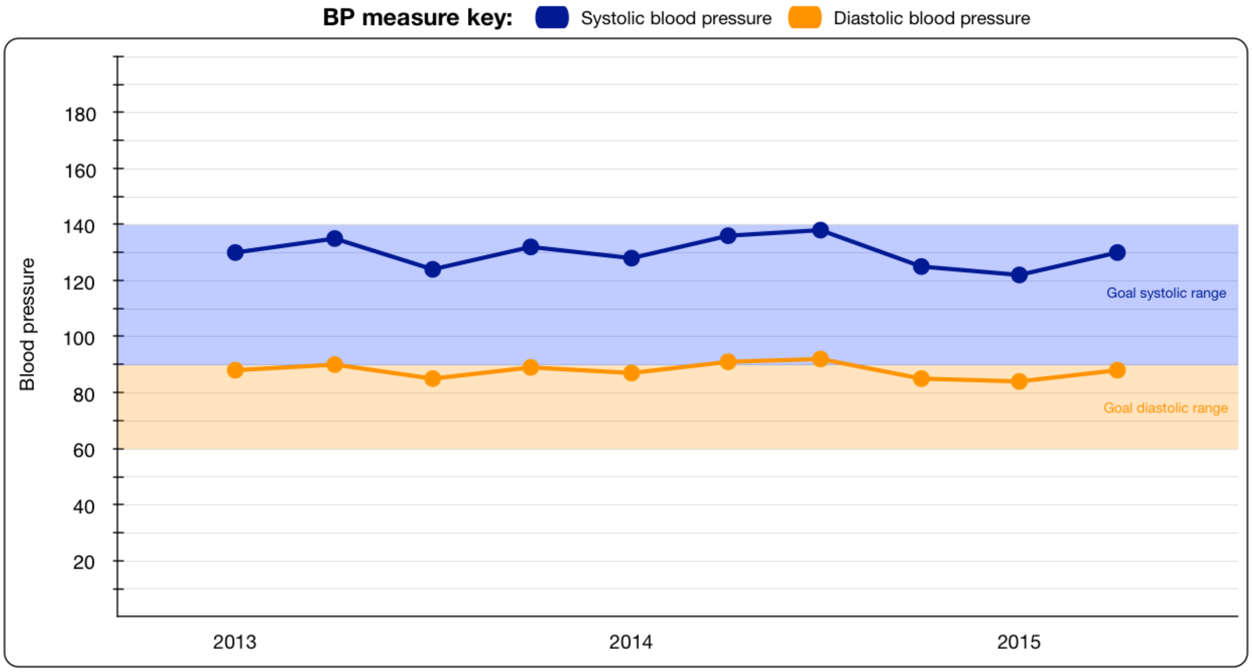

▽ Blood Pressure Values

**Systolic**	130	135	124	132	128	136	138	125	122	130
**Diastolic**	88	90	85	89	87	91	92	85	84	88

Vignette 2 (SBP Mean 130, SBP SD 15)

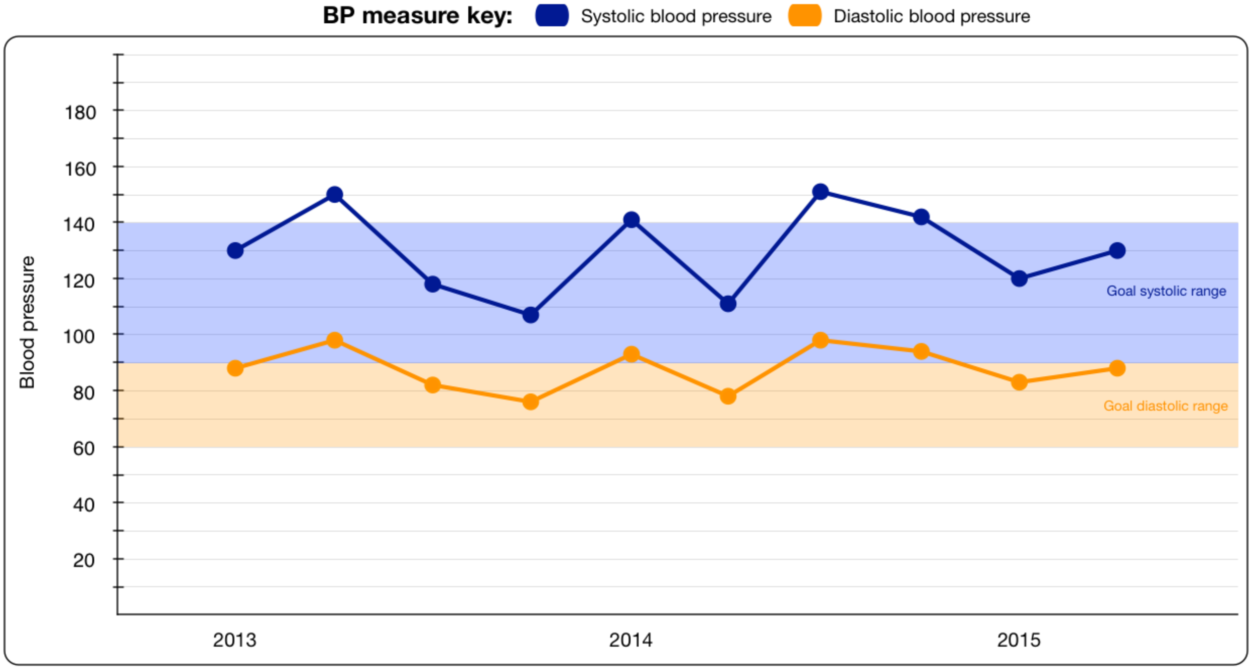

▽ Blood Pressure Values

**Systolic**	130	150	118	107	141	111	151	142	120	130
**Diastolic**	88	98	82	76	93	78	98	94	83	88

Vignette 3 (SBP Mean 130, SBP SD 25)

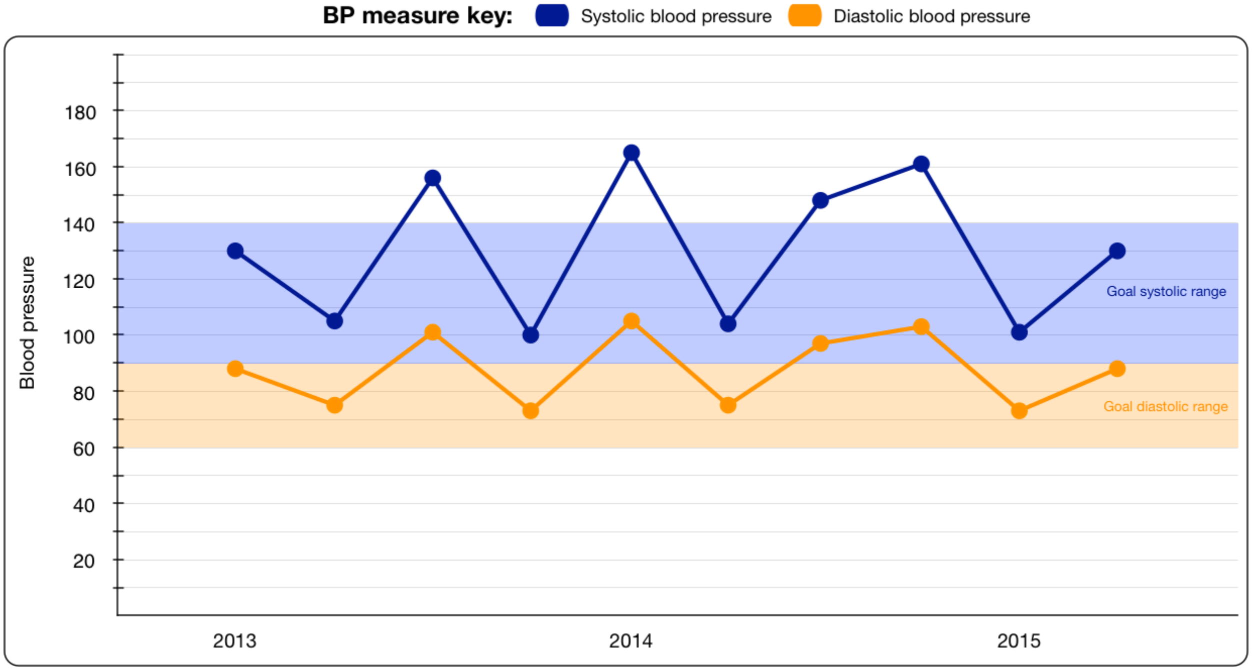

▽ Blood Pressure Values

**Systolic**	130	105	156	100	165	104	148	161	101	130
**Diastolic**	88	75	101	73	105	75	97	103	73	88

Vignette 4 (SBP Mean 145, SBP SD 5)

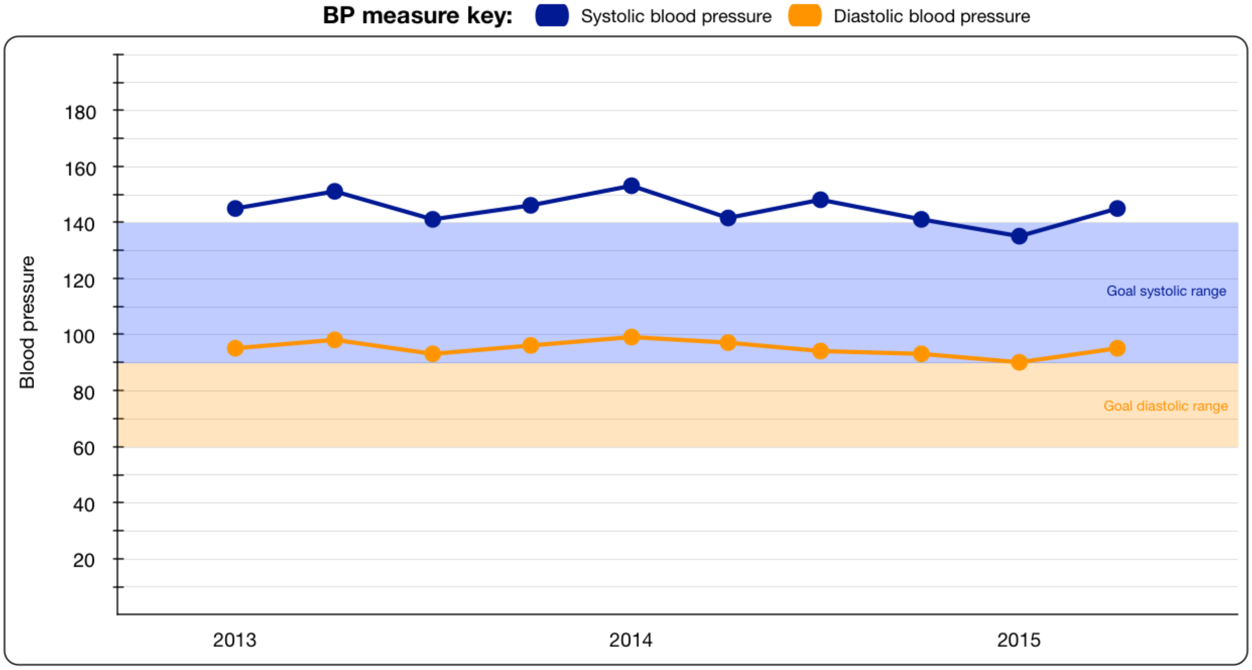

▽ Blood Pressure Values

**Systolic**	145	151	141	146	153	142	148	141	135	145
**Diastolic**	95	98	93	96	99	94	97	93	90	95

Vignette 5 (SBP Mean 145, SBP SD 15)

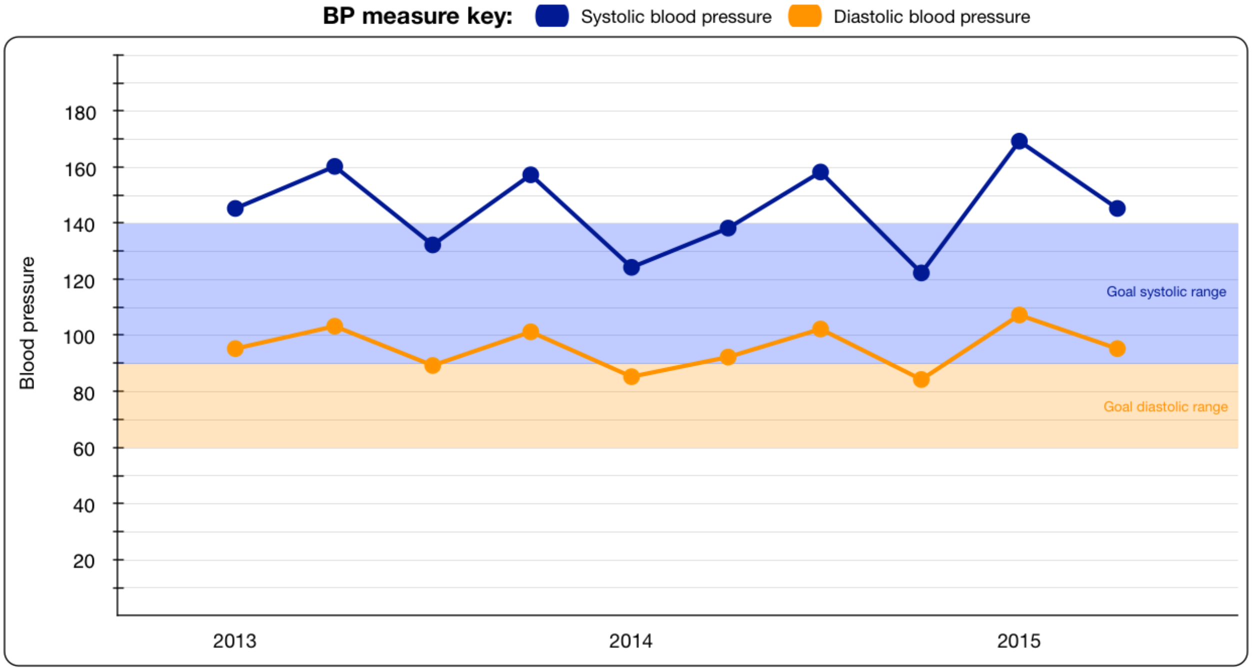

▽ Blood Pressure Values

**Systolic**	145	160	132	157	124	138	158	122	169	145
**Diastolic**	95	103	89	101	85	92	102	84	107	95

Vignette 6 (SBP Mean 145, SBP SD 25)

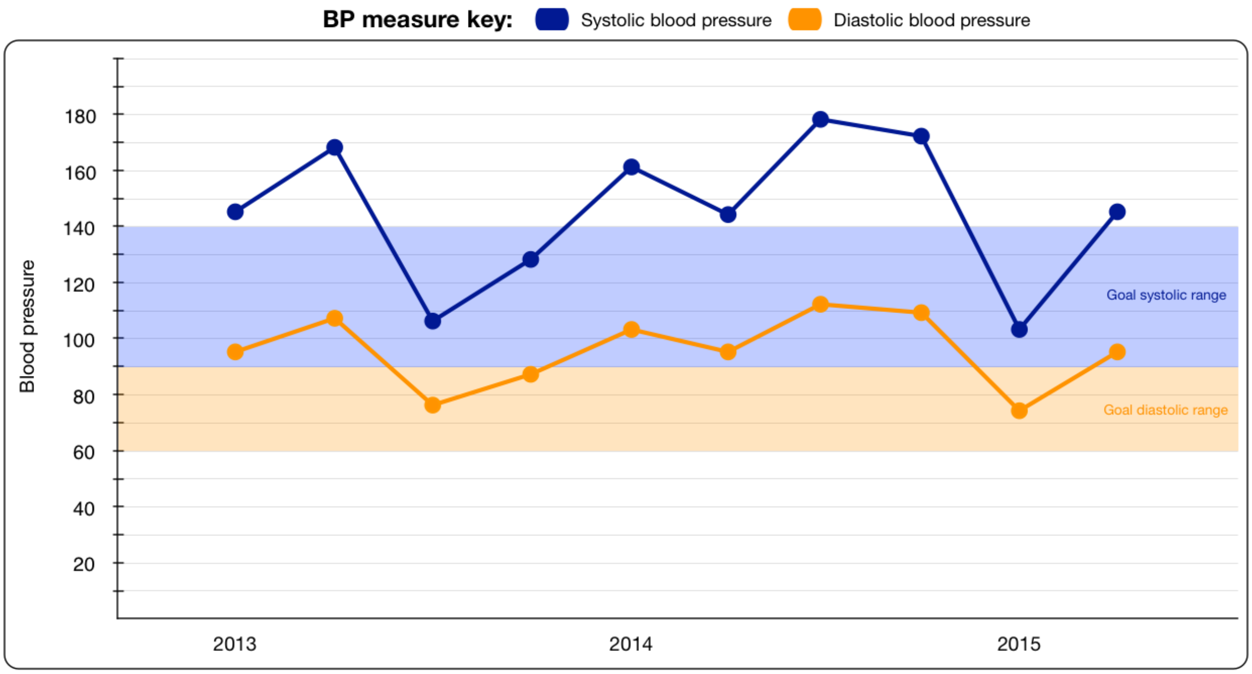

▽ Blood Pressure Values

**Systolic**	145	168	106	128	161	144	178	172	103	145
**Diastolic**	95	107	76	87	103	95	112	109	74	95

Vignette 7 (SBP Mean 160, SD 5)

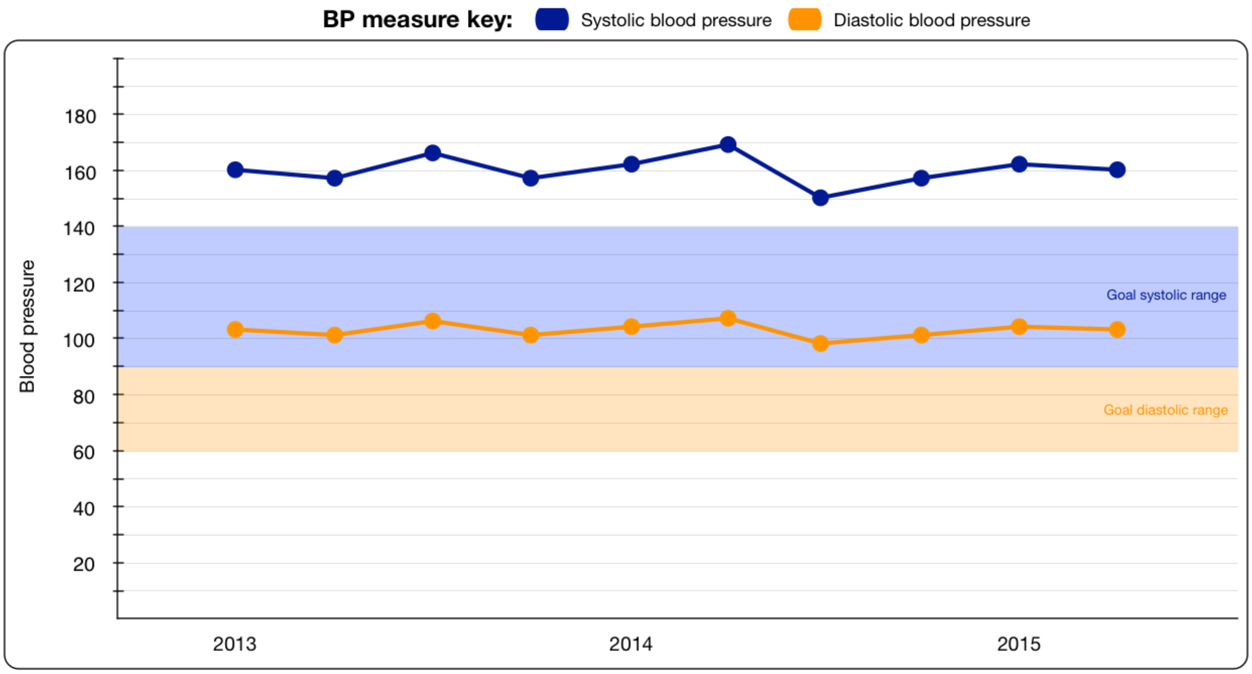

▽ Blood Pressure Values

**Systolic**	160	157	166	157	162	169	150	157	162	160
**Diastolic**	103	101	106	101	104	107	98	101	104	103

Vignette 8 (SBP Mean 160, SD 15)

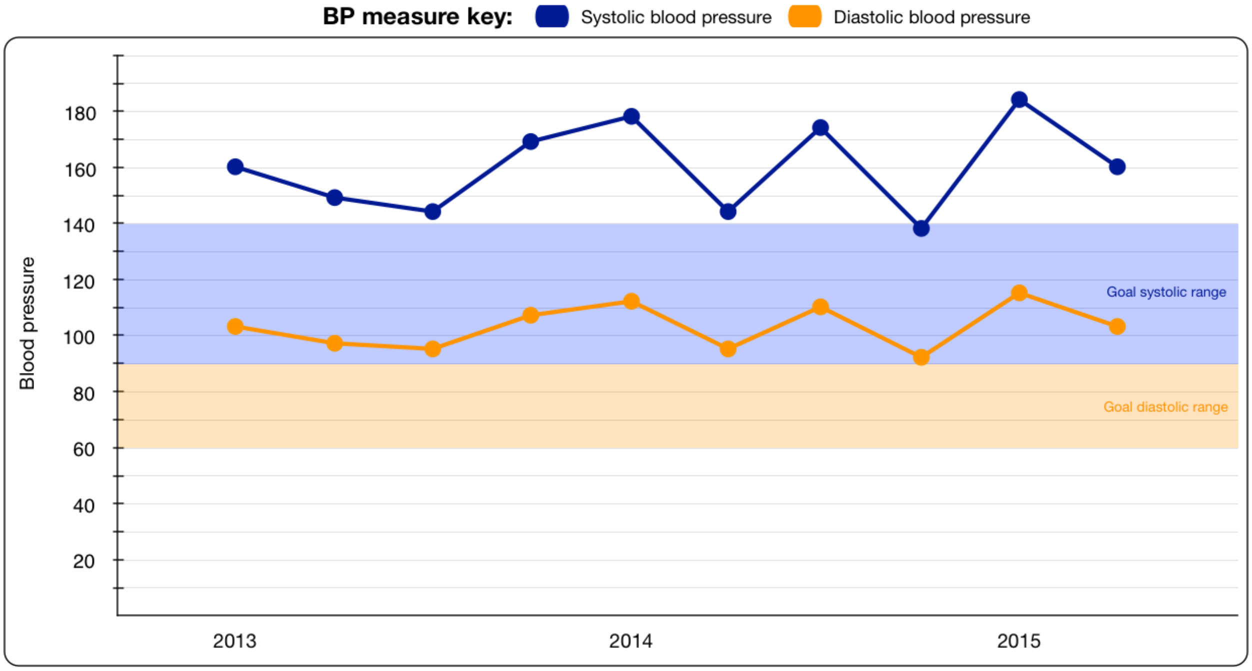

▽ Blood Pressure Values

**Systolic**	160	149	144	169	178	144	174	138	184	160
**Diastolic**	103	97	95	107	112	95	110	92	115	103

Vignette 9 (SBP Mean 160, SD 25)

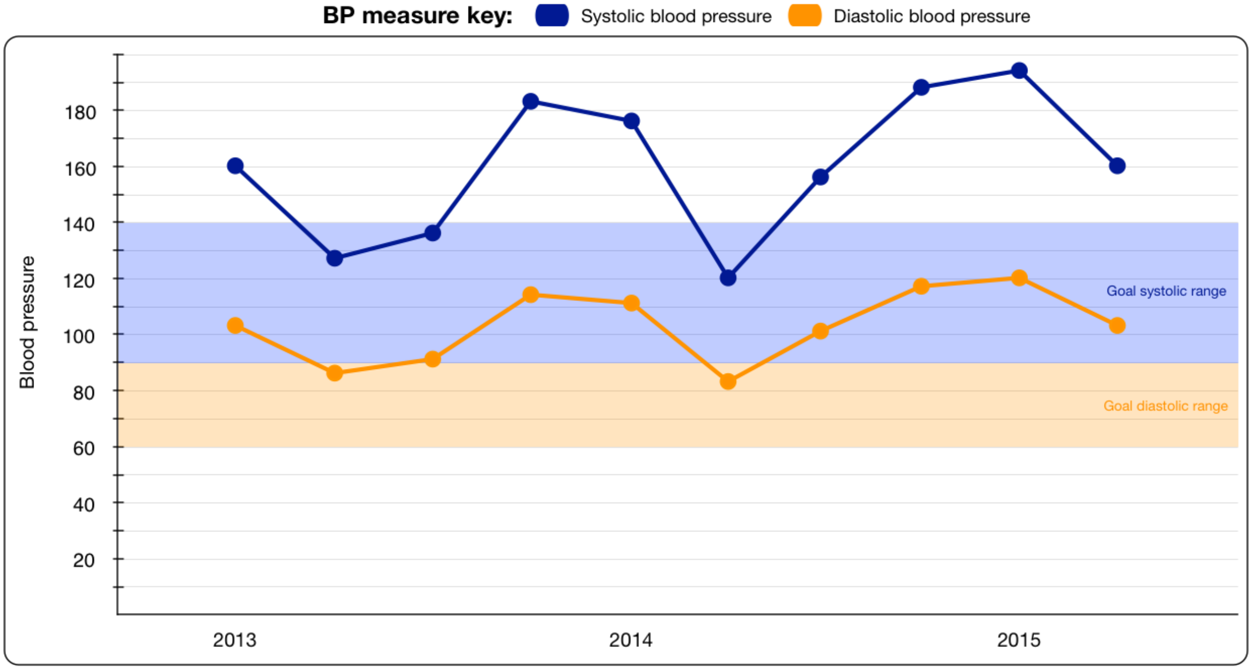

▽ Blood Pressure Values

**Systolic**	160	127	136	183	176	120	156	188	194	160
**Diastolic**	103	86	91	114	111	83	101	117	120	103
