# Supplementary material for: Patient Judgments About Hypertension Control: The Role of Variability, Trends, and Outliers in Visualized Blood Pressure Data
Source: J Med Internet Res. 2019 Mar 26;21(3):e11366. doi: 10.2196/11366 (PMC6454346; doi:10.2196/11366)
Supplement: Multimedia Appendix 2 [file jmir_v21i3e11366_app2.pdf]

Multimedia Appendix B: Study 2 Materials

Text for each vignette:

Mr. Brown visits his primary care physician and has his blood pressure measured. Mr. Brown and his doctor examine his blood pressure control over the last two years. Below is a graph showing his systolic and diastolic blood pressure over two years. After reviewing the graph, we will ask you several questions about Mr. Brown and his blood pressure data. For this task, you will need to know what normal values are for blood pressure. When you are resting, a normal systolic value (i.e. the top number) should be between 100 and 140 mmHg and a normal diastolic value (i.e. the bottom number) will be between 60 and 90mmHg. [The only variation in the text of the vignettes was the patient name. 6 total vignettes were presented in Study 2]

Vignette 1 (SBP Mean 130, Increasing slope)

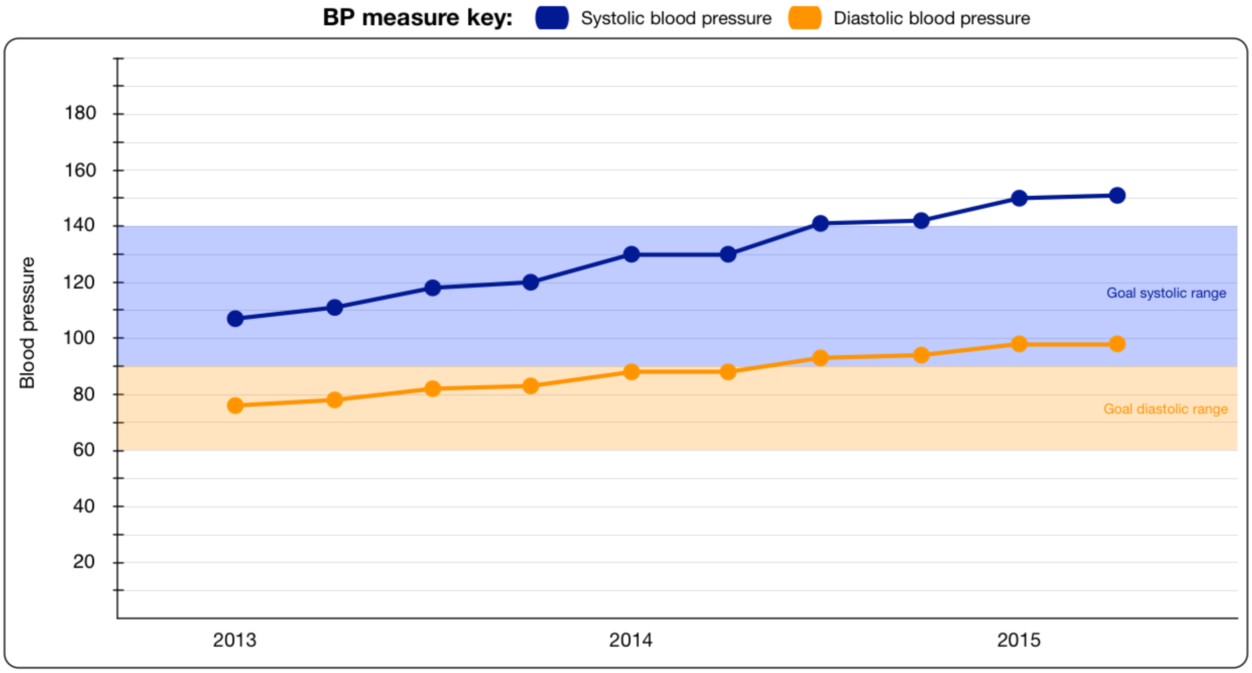

▽ Blood Pressure Values

**Systolic**	107	111	118	120	130	130	141	142	150	151
**Diastolic**	76	78	82	83	88	88	93	94	98	98

Vignette 2 (SBP Mean 130, Decreasing slope)

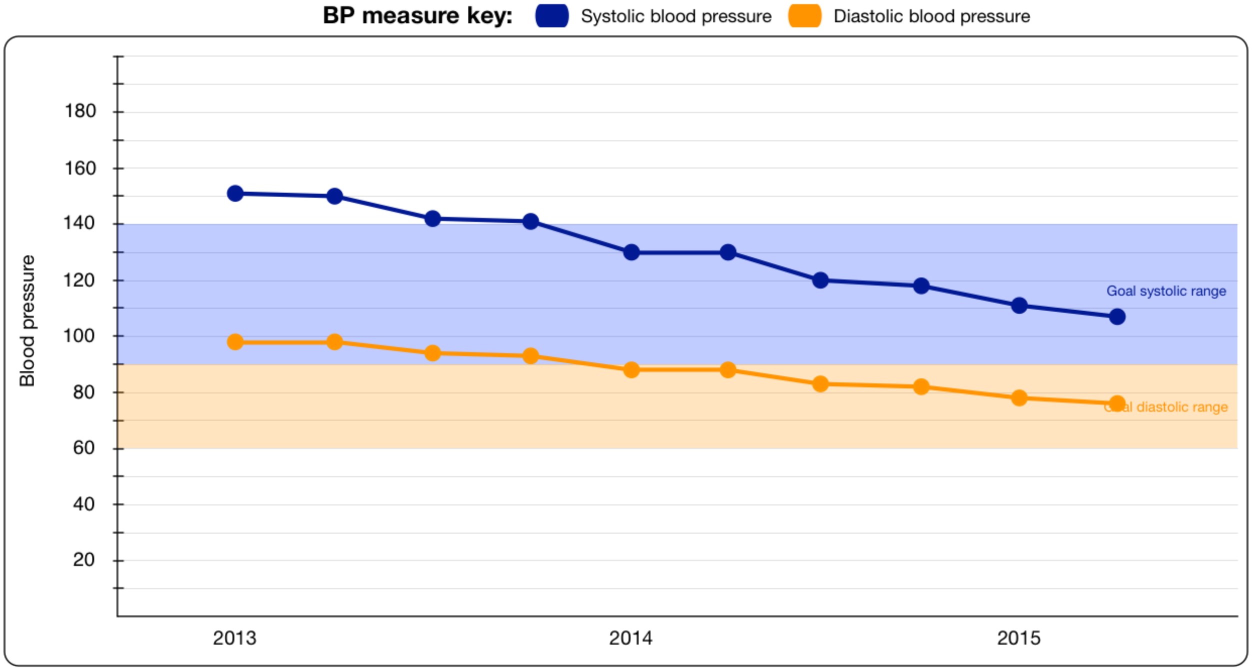

▽ Blood Pressure Values

**Systolic**	151	150	142	141	130	130	120	118	111	107
**Diastolic**	98	98	94	93	88	88	83	82	78	76

Vignette 3 (SBP Mean 145, Increasing slope)

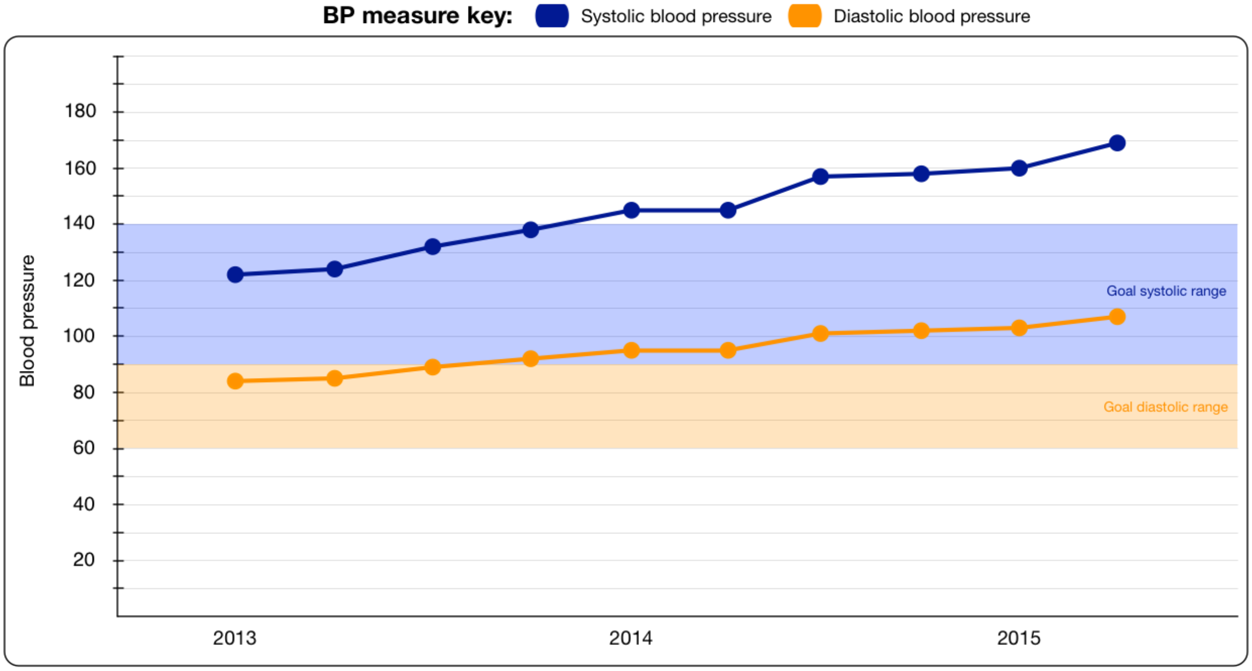

▽ Blood Pressure Values

**Systolic**	122	124	132	138	145	145	157	158	160	169
**Diastolic**	84	85	89	92	95	95	101	102	103	107

Vignette 4 (SBP Mean 145, Decreasing slope)

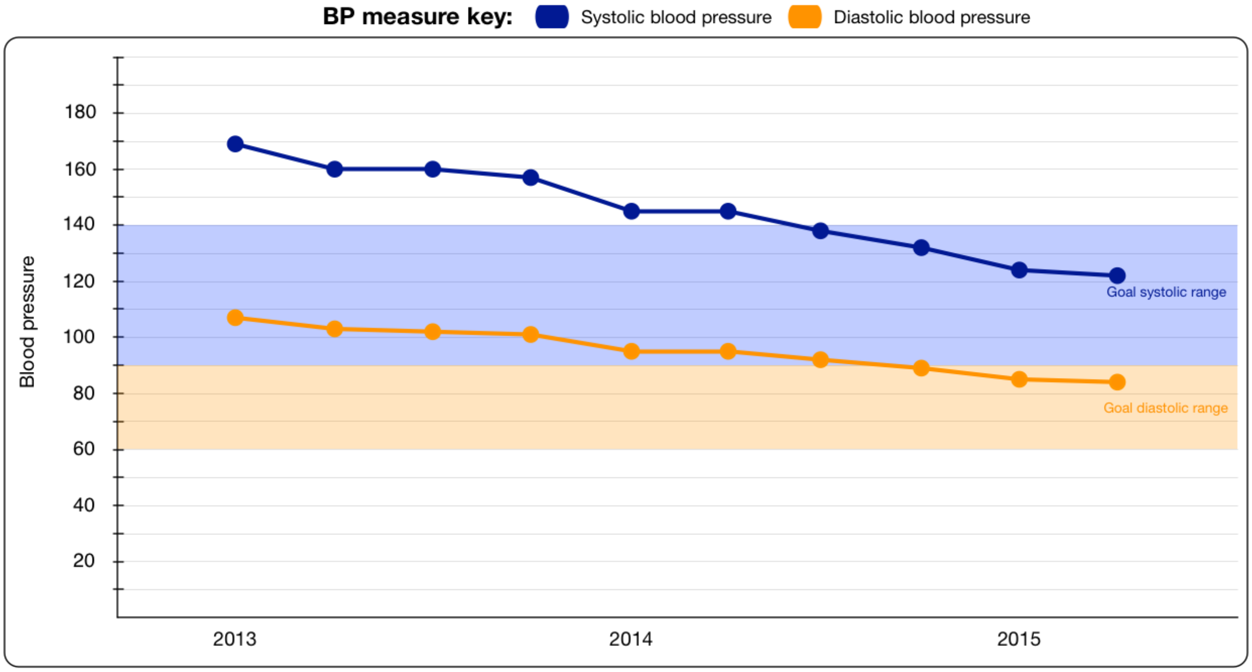

▽ Blood Pressure Values

**Systolic**	169	160	158	157	145	145	138	132	124	122
**Diastolic**	107	103	102	101	95	95	92	89	85	84

Vignette 5 (SBP Mean 160, Increasing slope)

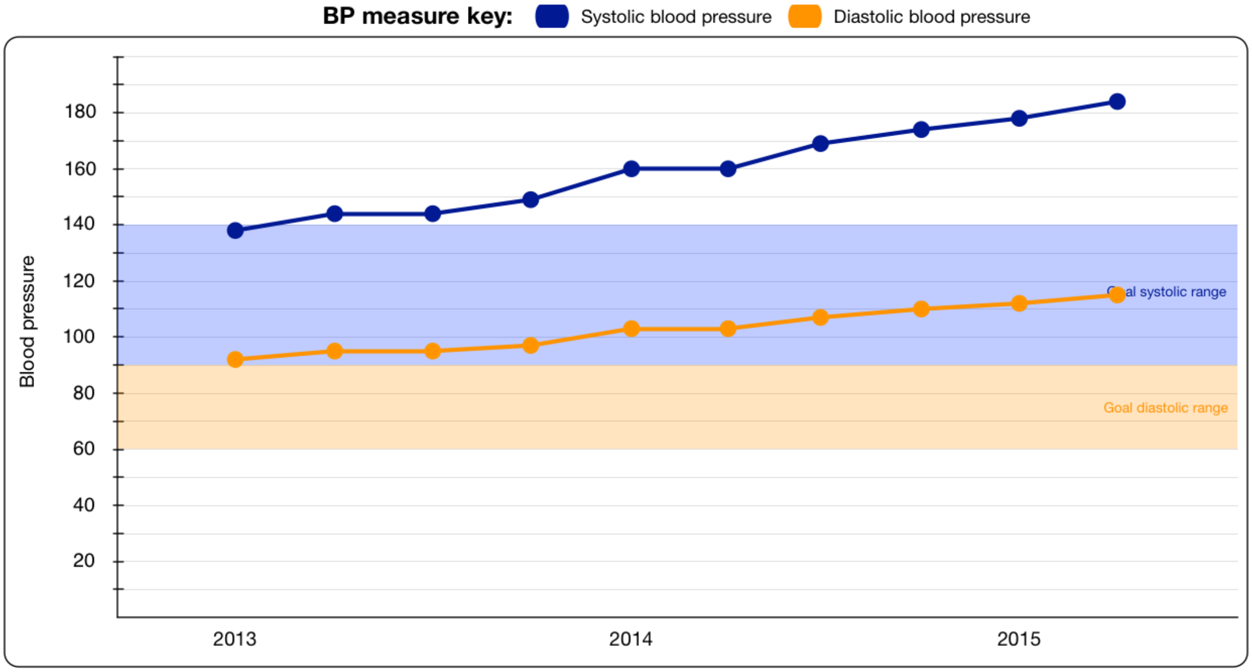

▽ Blood Pressure Values

**Systolic**	138	144	144	149	160	160	169	174	178	184
**Diastolic**	92	95	95	97	103	103	107	110	112	115

Vignette 6 (SBP Mean 160, Decreasing slope)

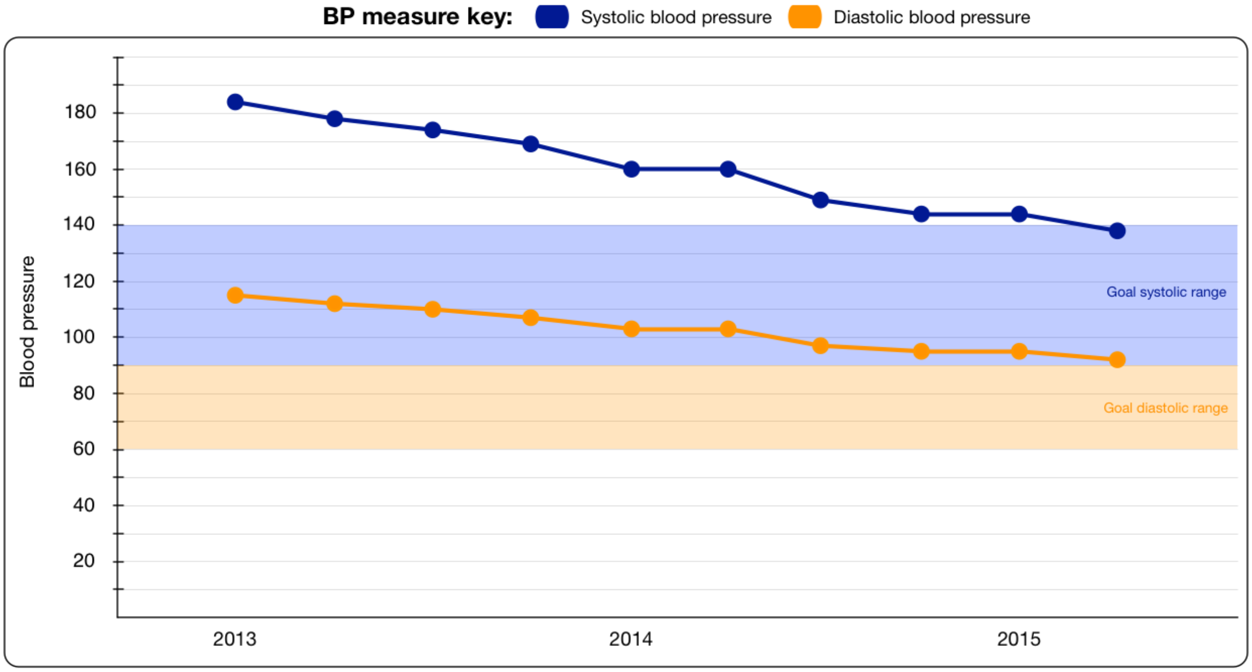

▽ Blood Pressure Values

**Systolic**	184	178	174	169	160	160	149	144	144	138
**Diastolic**	115	112	110	107	103	103	97	95	95	92
