# Supplementary material for: Patient Judgments About Hypertension Control: The Role of Variability, Trends, and Outliers in Visualized Blood Pressure Data
Source: J Med Internet Res. 2019 Mar 26;21(3):e11366. doi: 10.2196/11366 (PMC6454346; doi:10.2196/11366)
Supplement: Multimedia Appendix 3 [file jmir_v21i3e11366_app3.pdf]

Multimedia Appendix C: Study 3 Materials

Text for each vignette:

Mr. Brown visits his primary care physician and has his blood pressure measured. Mr. Brown and his doctor examine his blood pressure control over the last two years. Below is a graph showing his systolic and diastolic blood pressure over two years. After reviewing the graph, we will ask you several questions about Mr. Brown and his blood pressure data. For this task, you will need to know what normal values are for blood pressure. When you are resting, a normal systolic value (i.e. the top number) should be between 100 and 140 mmHg and a normal diastolic value (i.e. the bottom number) will be between 60 and 90mmHg. [The only variation in the text of the vignettes was the patient name. 10 total vignettes were presented in Study 3]

Vignette 1 (SBP Mean 130, No outliers)

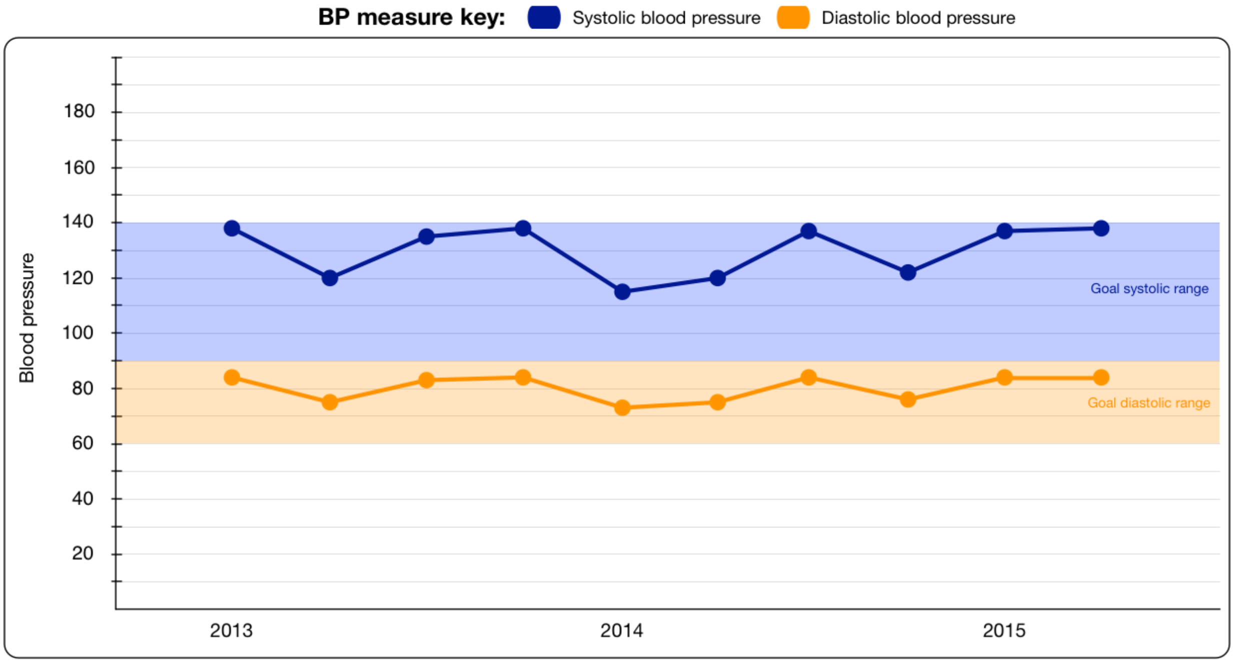

▽ Blood Pressure Values

**Systolic**	138	120	135	138	115	120	137	122	137	138
**Diastolic**	84	75	83	84	73	75	84	76	84	84

Vignette 2 (SBP Mean 130, 1 outlier above the mean)

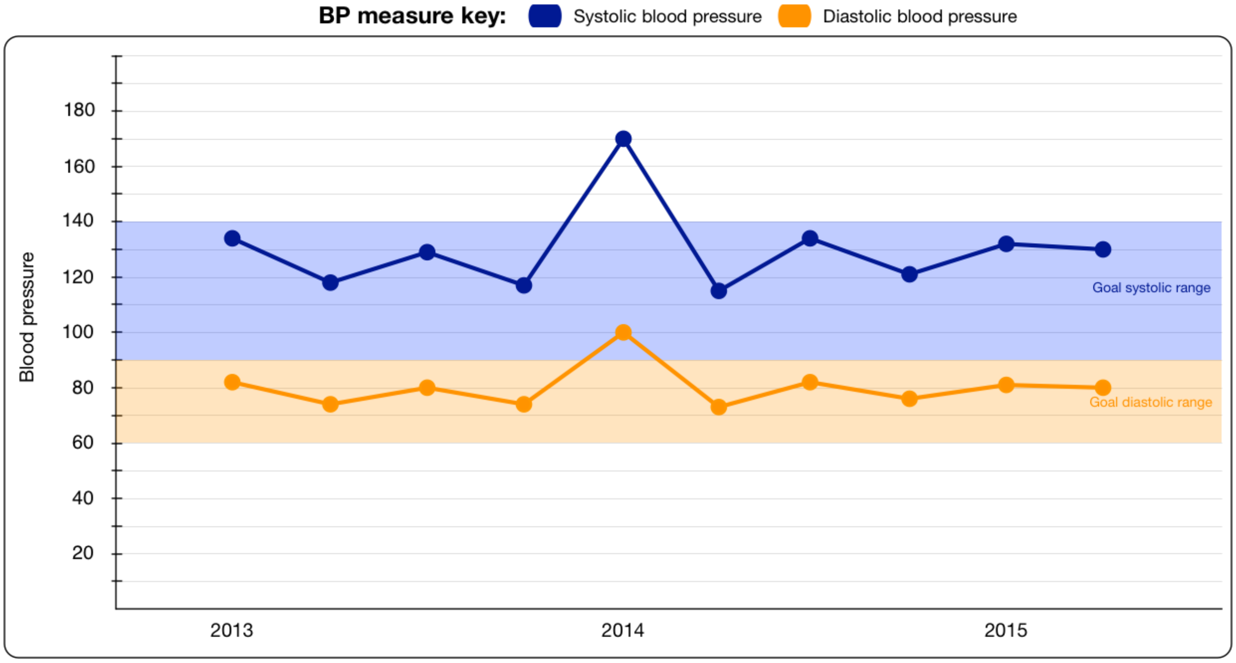

▽ Blood Pressure Values

**Systolic**	134	118	129	117	170	115	134	121	132	130
**Diastolic**	82	74	80	74	100	73	82	76	81	80

Vignette 3 (SBP Mean 130, 1 outlier below the mean)

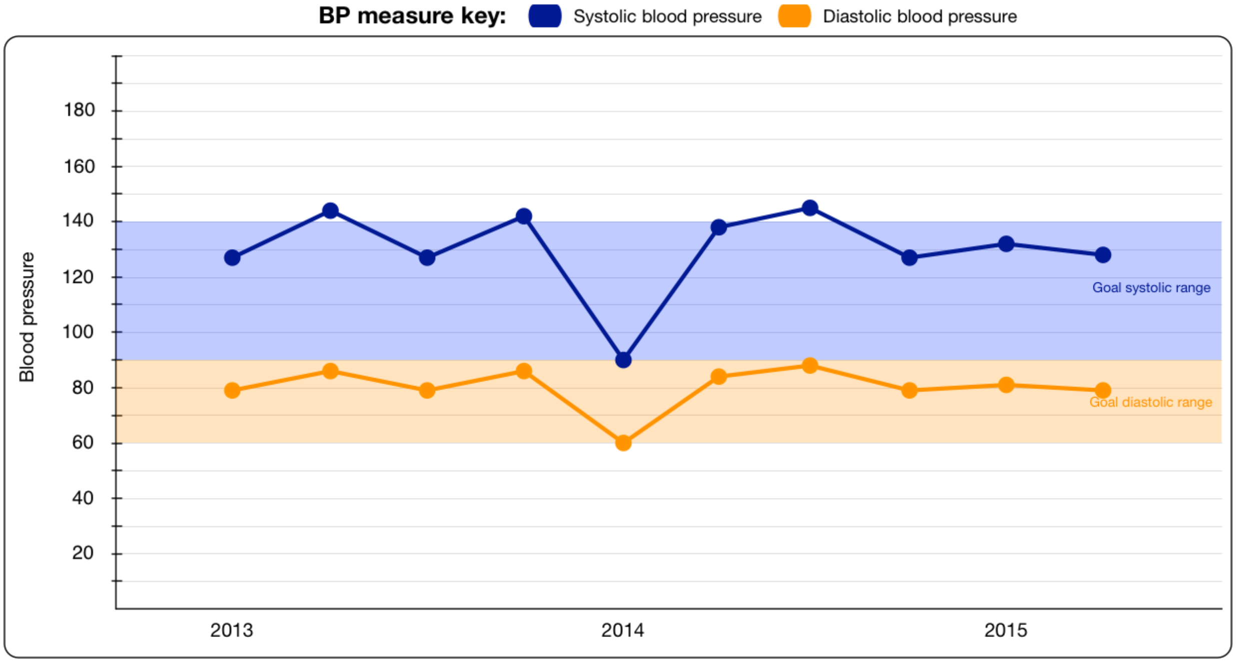

▽ Blood Pressure Values

**Systolic**	127	144	127	142	90	138	145	127	132	128
**Diastolic**	79	87	79	86	60	84	88	79	81	79

Vignette 4 (SBP Mean 130, 2 outliers above the mean)

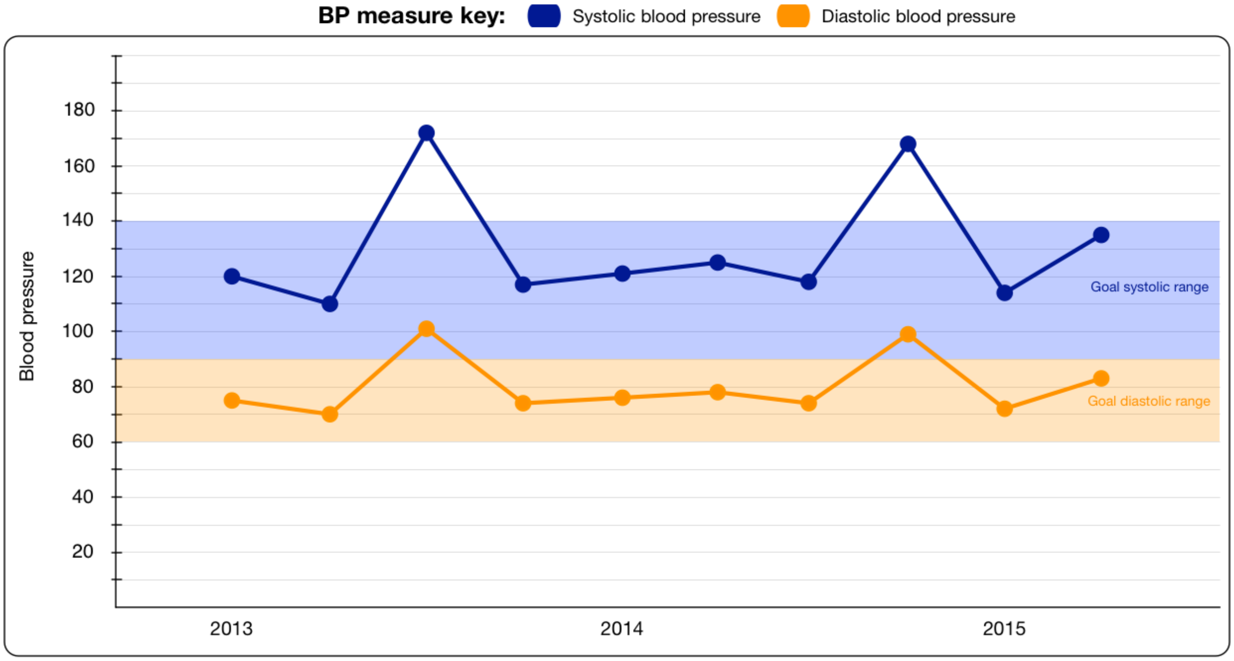

Blood Pressure Values

**Systolic**	120	110	172	117	121	125	118	168	114	135
**Diastolic**	75	70	101	74	76	78	74	99	72	83

Vignette 5 (SBP Mean 130, 2 outliers below the mean)

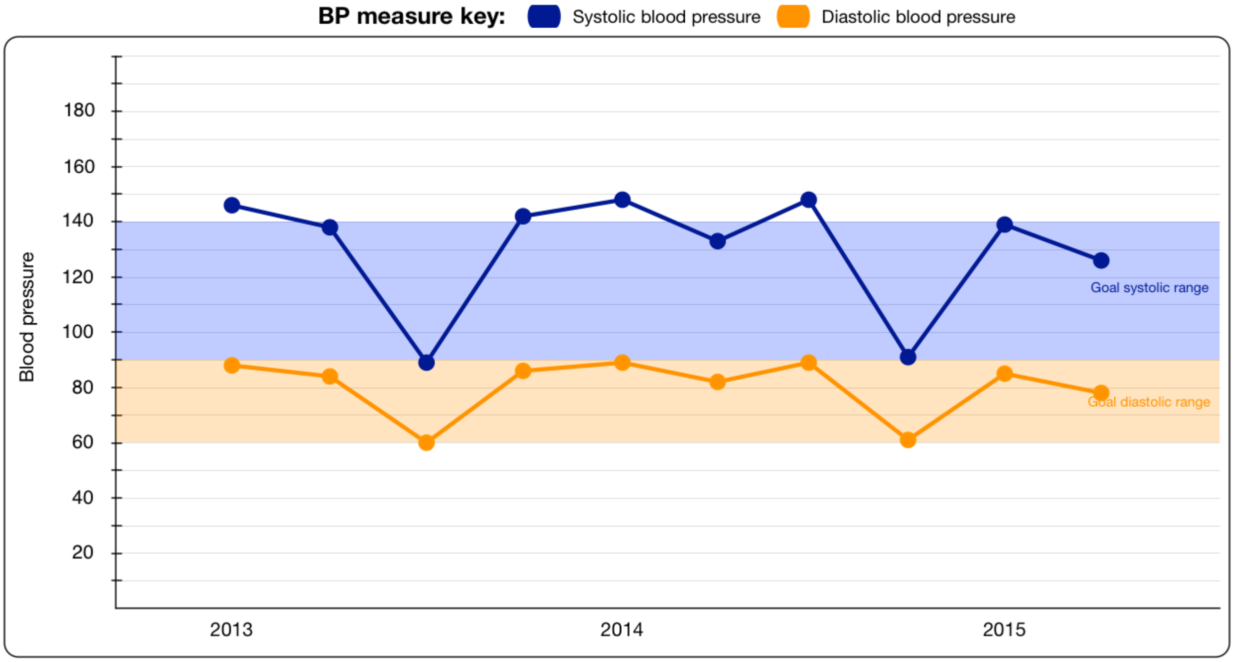

Blood Pressure Values

**Systolic**	146	138	89	142	148	133	148	91	139	126
**Diastolic**	88	84	60	86	89	82	89	61	85	78

Vignette 6 (SBP Mean 145, no outliers)

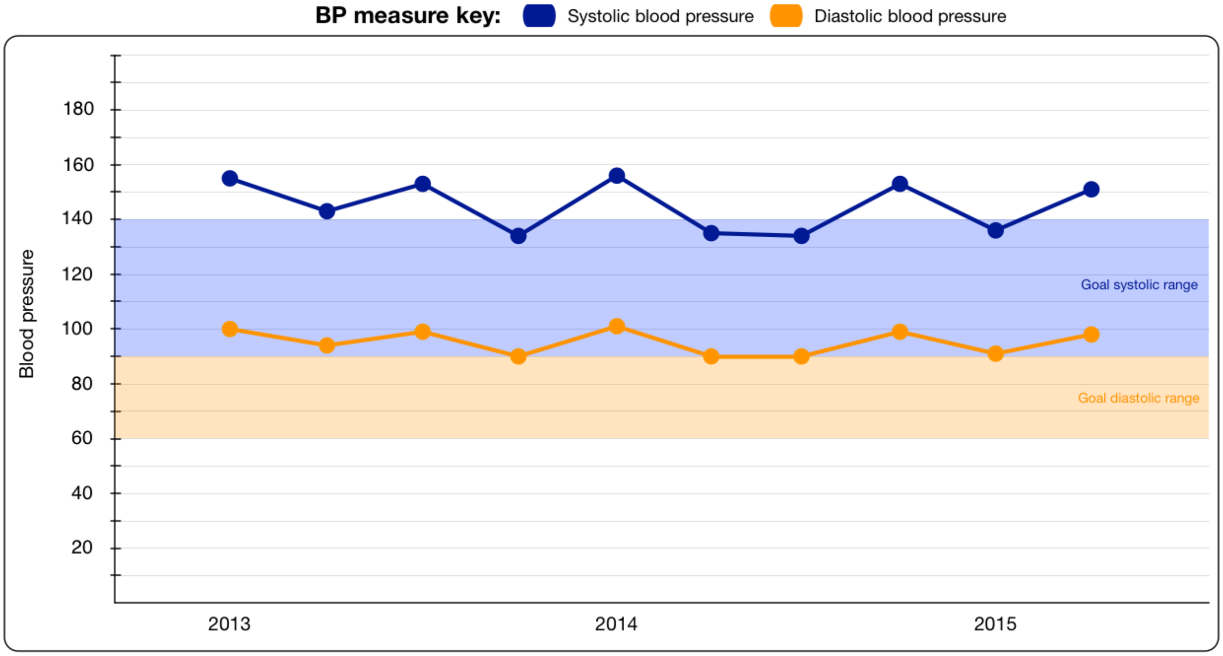

▽ Blood Pressure Values

**Systolic**	155	143	153	134	156	135	134	153	136	151
**Diastolic**	100	94	99	90	101	90	90	99	91	98

Vignette 7 (SBP Mean 145, 1 outlier above the mean)

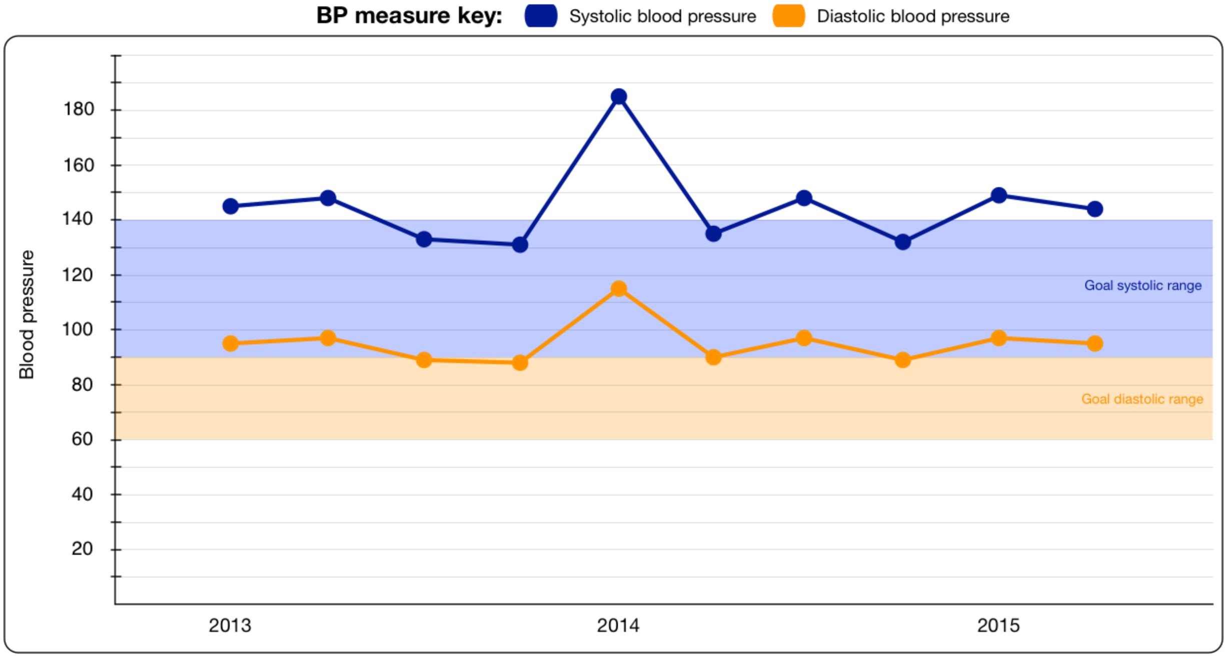

▽ Blood Pressure Values

**Systolic**	145	148	133	131	185	135	148	132	149	144
**Diastolic**	95	97	89	88	115	90	97	89	97	95

Vignette 8 (SBP Mean 145, 1 outlier below the mean)

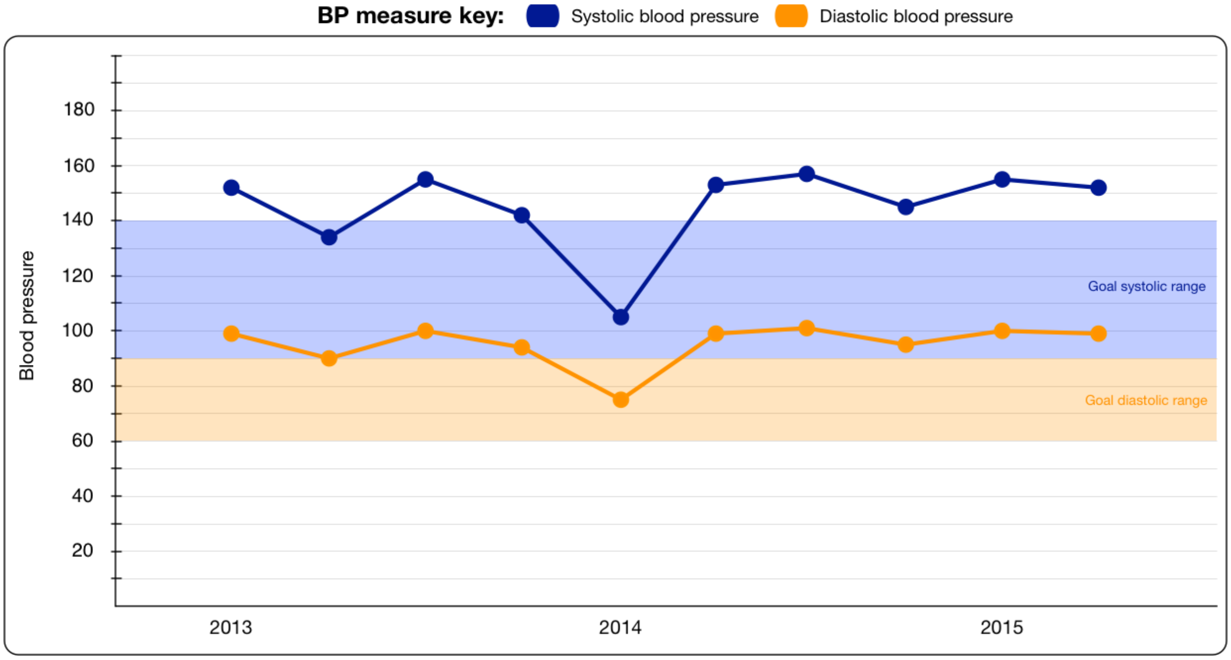

▽ Blood Pressure Values

**Systolic**	152	134	155	142	105	153	157	145	155	152
**Diastolic**	99	90	100	94	75	99	101	95	100	99

Vignette 9 (SBP Mean 145, 2 outliers above the mean)

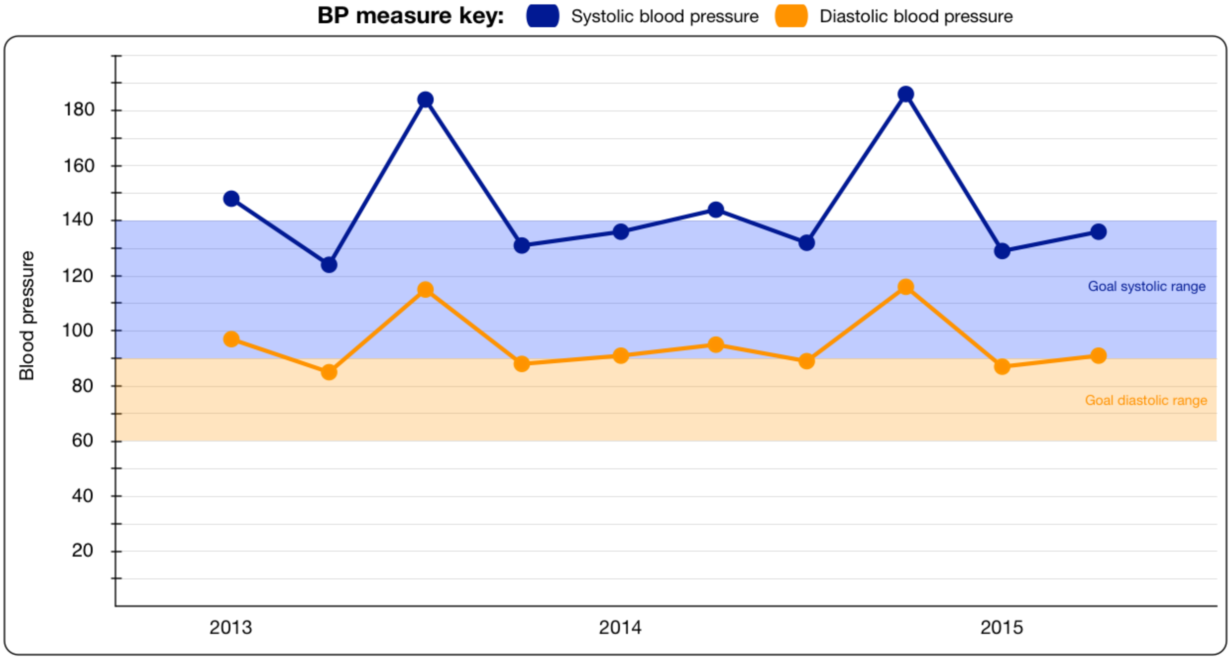

▽ Blood Pressure Values

**Systolic**	148	124	184	131	136	144	132	186	129	136
**Diastolic**	97	85	115	88	91	95	89	116	87	91

Vignette 10 (SBP Mean 145, 2 outliers below the mean)

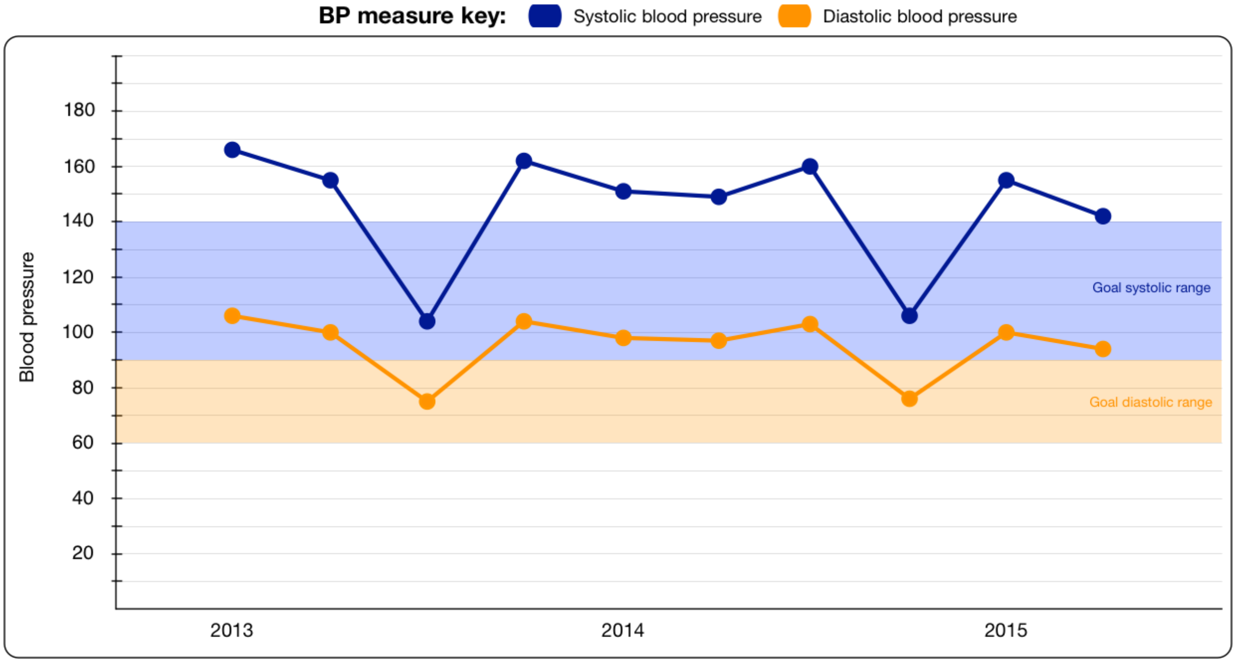

▽ Blood Pressure Values

**Systolic**	166	155	104	162	151	149	160	106	155	142
**Diastolic**	106	100	75	104	98	97	103	76	100	94
