# Supplementary material for: Patient Judgments About Hypertension Control: The Role of Variability, Trends, and Outliers in Visualized Blood Pressure Data
Source: J Med Internet Res. 2019 Mar 26;21(3):e11366. doi: 10.2196/11366 (PMC6454346; doi:10.2196/11366)
Supplement: Multimedia Appendix 4 [file jmir_v21i3e11366_app4.pdf]

Multimedia Appendix D: Results by Gender

Level of agreement with the following statements (0 'Strongly Disagree'—100 'Strongly Agree')	SD = 5	SD = 15	SD = 25
This patient needs to change their medication			
Mean BP = 130	27.579 (14.34, 40.82)	44.737 (32.46, 57.02)	72.368 (59.85, 84.89)
Mean BP = 145	57.000 (43.31, 70.69)	78.632 (67.05, 90.22)	85.526 (78.32, 92.74)
Mean BP = 160	83.105 (70.66, 95.55)	83.579 (71.66, 95.5)	81.421 (68.76, 94.08)
This patient's blood pressure is well controlled			
Mean BP=130	81.789 (72.41, 91.17)	62.421 (51.7, 73.14)	29.526 (17.76, 41.29)
Mean BP=145	45.947 (32.83, 59.06)	19.947 (9.54, 30.36)	21.947 (9.28, 34.62)
Mean BP=160	25.579 (11.66, 39.5)	19.053 (5.69, 32.41)	14.211 (4.98, 23.44)
Perceived likelihood of the following events (1 'Extremely Unlikely'–10 'Extremely Likely') *Heart attack in the next 10 years*			
Mean BP=130	3.211 (2.33, 4.09)	3.526 (2.74, 4.31)	6.368 (5.38, 7.35)
Mean BP=145	5.842 (4.7, 6.99)	6.421 (5.18, 7.66)	7.105 (6.26, 7.95)
Mean BP=160	7.421 (6.2, 8.64)	7.895 (6.83, 8.96)	7.947 (7.04, 8.85)
Stroke in the next 10 years			
Mean BP=130	3.158 (2.36, 3.95)	3.737 (2.81, 4.66)	6.579 (5.49, 7.66)
Mean BP=145	5.737 (4.6, 6.88)	6.632 (5.53, 7.74)	7.474 (6.73, 8.21)
Mean BP=160	7.737 (6.56, 8.92)	7.947 (6.88, 9.01)	8.263 (7.42, 9.11)

Experiment 1. Results—Males , M (95% CI)

Level of agreement with the following statements (0 ‘Strongly Disagree’—100 ‘Strongly Agree’)	SD = 5	SD = 15	SD = 25
This patient needs to change their medication			
Mean BP = 130	31.062 (20.57, 41.55)	48.969 (38.91, 59.03)	70.625 (60.57, 80.68)
Mean BP = 145	58.844 (47.74, 69.95)	75.281 (66.05, 84.51)	84.75 (77.04, 92.46)
Mean BP = 160	88.469 (82.82, 94.12)	86.25 (78.37, 94.13)	84.625 (75.96, 93.29)
This patient’s blood pressure is well controlled			
Mean BP=130	77.938 (70.4, 85.47)	53.5 (42.94, 64.06)	28.969 (19.09, 38.85)
Mean BP=145	35.781 (24.65, 46.92)	25.531 (15.62, 35.44)	21.719 (11.74, 31.69)
Mean BP=160	21.062 (11.15, 30.98)	15.156 (7.19, 23.12)	19.562 (9.6, 29.53)
Perceived likelihood of the following events (1 ‘Extremely Unlikely’–10 ‘Extremely Likely’) *Heart attack in the next 10 years*			
Mean BP=130	4.188 (3.5, 4.87)	4.75 (3.93, 5.57)	6.312 (5.56, 7.07)
Mean BP=145	5.438 (4.61, 6.27)	7.688 (7.05, 8.32)	7.906 (7.28, 8.53)
Mean BP=160	7.812 (7.17, 8.46)	8.031 (7.21, 8.85)	8.219 (7.72, 8.72)
Stroke in the next 10 years			
Mean BP=130	4.125 (3.34, 4.91)	4.875 (3.99, 5.76)	6.656 (5.81, 7.50)
Mean BP=145	5.781 (4.95, 6.61)	7.906 (7.29, 8.52)	8.312 (7.8, 8.83)
Mean BP=160	8.219 (7.63, 8.81)	8.25 (7.46, 9.04)	8.406 (7.93, 8.89)

Experiment 1. Results—Females , M (95% CI)

Level of agreement with the following statements (0 ‘Strongly Disagree’–100 ‘Strongly Agree’)	Increasing	Decreasing
This patient’s blood pressure is well controlled		
Mean BP = 130	39.000<br>(25.75, 52.25)	81.412<br>(69.73, 93.1)
Mean BP = 145	24.353<br>(10.93, 37.78)	75.765<br>(64.03, 87.5)
Mean BP = 160	20.412<br>(4.14, 36.69)	45.118<br>(29.08, 61.16)
This patient needs to change their medication		
Mean BP=130	82.765<br>(77.06, 88.47)	27.588<br>(10.78, 44.39)
Mean BP=145	84.647<br>(74.11, 95.19)	28.529<br>(14.63, 42.42)
Mean BP=160	89.882<br>(79.93, 99.83)	49.882<br>(33.39, 66.37)
Perceived likelihood of the following events (1 ‘Extremely Unlikely’–10 ‘Extremely Likely)		
Heart attack in the next 10 years		
Mean BP=130	6.647<br>(5.76, 7.54)	3.353<br>(2.21, 4.49)
Mean BP=145	7.588<br>(6.8, 8.38)	3.529<br>(2.39, 4.67)
Mean BP=160	8.176<br>(7.18, 9.17)	5.529<br>(4.36, 6.7)
Stroke in the next 10 years		
Mean BP=130	6.706<br>(5.65, 7.77)	3.118<br>(1.92, 4.32)
Mean BP=145	7.176<br>(6.02, 8.33)	3.412<br>(2.31, 4.51)
Mean BP=160	8.235<br>(7.18, 9.29)	5.353<br>(4.01, 6.70)
SBP Recall		
Mean BP=130	59.588<br>(47.8, 71.37)	35.706<br>(20.67, 50.74)
Mean BP=145	62.706<br>(49.63, 75.79)	46.941<br>(33.25, 60.63)
Mean BP=160	80.059<br>(68.46, 91.65)	59.000<br>(44.13, 73.87)

Experiment 2. Results—Males, M (95% CI)

Level of agreement with the following statements (0 ‘Strongly Disagree’–100 ‘Strongly Agree’)	Increasing	Decreasing
This patient’s blood pressure is well controlled		
Mean BP = 130	26.697 (15.5, 37.9)	87.939 (80.39, 95.49)
Mean BP = 145	18.364 (9.94, 26.79)	72.788 (63.17, 82.41)
Mean BP = 160	6.545 (2.9, 10.19)	52.303 (41.02, 63.58)
This patient needs to change their medication		
Mean BP=130	82 .000 (72.92, 91.08)	18.667 (10.1, 27.23)
Mean BP=145	86.364 (78.49, 94.24)	26.697 (17.3, 36.1)
Mean BP=160	91.273 (82.78, 99.76)	43.242 (31.44, 55.04)
Perceived likelihood of the following events (1 ‘Extremely Unlikely’–10 ‘Extremely Likely)		
Heart attack in the next 10 years		
Mean BP=130	7.455 (6.71, 8.2)	2.848 (2.18, 3.52)
Mean BP=145	8.152 (7.42, 8.88)	3.727 (2.96, 4.49)
Mean BP=160	9.424 (9.1, 9.74)	5.636 (4.75, 6.52)
Stroke in the next 10 years		
Mean BP=130	7.424 (6.68, 8.17)	2.879 (2.2, 3.56)
Mean BP=145	8.394 (7.76, 9.03)	3.697 (2.97, 4.42)
Mean BP=160	9.515 (9.13, 9.9)	5.667 (4.77, 6.56)
SBP Recall		
Mean BP=130	54.879 (46.1, 63.66)	26.152 (17.34, 34.96)
Mean BP=145	64.455 (56.51, 72.4)	43.727 (34.72, 52.73)
Mean BP=160	87.03 (81.06, 93)	63.212 (51.23, 75.2)

Experiment 2. Results—Females, M (95% CI)

	1 Down	1 Up	2 Down	2 Up	Uniform
Level of agreement with the following statements (0 'Strongly Disagree'–100 'Strongly Agree)					
This patient's blood pressure is well controlled					
Mean BP=130	70.333 (58.55, 82.11)	72.667 (62.57, 82.76)	45.833 (30.58, 61.08)	42.500 (28.81, 56.19)	90.611 (83.64, 97.58)
Mean BP=145	21.778 (10.32, 33.23)	33.5 (20.55, 46.45)	15.278 (4.1, 26.45)	25.333 (12.98, 37.69)	33.5 (17.9, 49.1)
This patient needs to change their medication					
Mean BP=130	32.222 (19.58, 44.87)	29.278 (17.71, 40.85)	43.111 (28.98, 57.25)	51.722 (36.9, 66.54)	14.722 (3.32, 26.13)
Mean BP=145	75.778 (63.09, 88.46)	63.944 (48.14, 79.75)	82.611 (68.65, 96.57)	80.278 (67.3, 93.26)	64.889 (47.45, 82.33)
Perceived likelihood of the following events (1 'Extremely Unlikely'–10 'Extremely Likely')					
Heart attack in the next 10 years					
Mean BP=130	3.944 (2.86, 5.02)	3.611 (2.76, 4.47)	4.500 (3.4, 5.6)	5.333 (4.19, 6.48)	2.278 (1.78, 2.77)
Mean BP=145	7.056 (5.95, 8.16)	6.278 (5.16, 7.4)	7.556 (6.39, 8.72)	7.611 (6.39, 8.83)	6.667 (5.34, 7.99)
Stroke in the next 10 years					
Mean BP=130	4.056 (2.91, 5.2)	3.667 (2.8, 4.53)	4.500 (3.4, 5.6)	5.167 (4, 6.33)	2.278 (1.73, 2.82)
Mean BP=145	7.333 (6.21, 8.45)	6.556 (5.42, 7.69)	7.889 (6.73, 9.05)	7.667 (6.43, 8.9)	6.667 (5.27, 8.07)
% of SBP points out of range (0–100%)					
Mean BP=130	25.778 (11.9, 39.66)	21.722 (9.16, 34.29)	38.056 (23.79, 52.32)	31.389 (20.55, 42.23)	28.722 (10.34, 47.11)
Mean BP=145	56.0000 (41.87, 70.13)	48.056 (35.39, 60.72)	56.333 (41.58, 71.09)	52.056 (38.98, 65.13)	59.111 (44.91, 73.31)

Experiment 3. Results—Males, M(95% CI)

	1 Down	1 Up	2 Down	2 Up	Uniform
Level of agreement with the following statements (0 'Strongly Disagree'–100 'Strongly Agree)					
This patient's blood pressure is well controlled					
Mean BP=130	66.278 (55.42, 77.14)	67.081 (57.32, 76.84)	47.595 (37.72, 57.47)	58.216 (47.92, 68.51)	88.541 (81.9, 95.18)
Mean BP=145	31.757 (21.35, 42.16)	44.135 (34.01, 54.26)	20.946 (11.87, 30.02)	46.054 (35.26, 56.85)	38.757 (28.2, 49.31)
This patient needs to change their medication					
Mean BP=130	32.194 (22.21,	35.081 (24.37,	59.27 (51.14, 67.4)	48.973 (37.97,	23.865

	42.18)	45.79)		59.98)	(13.35, 34.38)
Mean BP=145	77.486 (68.54, 86.43)	65.568 (55.24, 75.89)	85.432 (78.52, 92.34)	69.838 (60.45, 79.23)	73.865 (65.01, 82.72)
Perceived likelihood of the following events (1 ‘Extremely Unlikely’–10 ‘Extremely Likely’)					
Heart attack in the next 10 years					
Mean BP=130	3.806 (3.1, 4.51)	4.216 (3.29, 5.15)	5.027 (4.27, 5.78)	5.27 (4.38, 6.16)	2.838 (2.11, 3.57)
Mean BP=145	7.081 (6.4, 7.76)	6.216 (5.52, 6.91)	7.757 (7.12, 8.39)	6.432 (5.58, 7.28)	6.297 (5.57, 7.03)
Stroke in the next 10 years					
Mean BP=130	3.722 (3.01, 4.44)	4.243 (3.35, 5.14)	5.027 (4.22, 5.83)	5.459 (4.55, 6.37)	2.811 (2.14, 3.49)
Mean BP=145	7.351 (6.62, 8.08)	6.351 (5.68, 7.02)	8.027 (7.37, 8.69)	6.595 (5.74, 7.45)	6.486 (5.76, 7.21)
% of SBP points out of range (0–100%)					
Mean BP=130	31.139 (22.49, 39.79)	32.243 (22.1, 42.38)	43.297 (34.25, 52.34)	43.405 (34.01, 52.8)	15.135 (7.58, 22.69)
Mean BP=145	64.595 (56.13, 73.06)	57.027 (48.58, 65.47)	70.568 (62.65, 78.48)	53.73 (44.78, 62.68)	59.432 (51.03, 67.84)

Experiment 3. Results—Females, M(95% CI)
